# Single-cell immunophenotyping revealed the association of CD4+ central and CD4+ effector memory T cells linking exacerbating chronic obstructive pulmonary disease and NSCLC

**DOI:** 10.3389/fimmu.2023.1297577

**Published:** 2023-12-20

**Authors:** Nikolett Gémes, József Á. Balog, Patrícia Neuperger, Erzsébet Schlegl, Imre Barta, János Fillinger, Balázs Antus, Ágnes Zvara, Zoltán Hegedűs, Zsolt Czimmerer, Máté Manczinger, Gergő Mihály Balogh, József Tóvári, László G. Puskás, Gábor J. Szebeni

**Affiliations:** ^1^ Laboratory of Functional Genomics, HUN-REN Biological Research Centre, Szeged, Hungary; ^2^ PhD School in Biology, University of Szeged, Szeged, Hungary; ^3^ National Korányi Institute of Pulmonology, Budapest, Hungary; ^4^ Laboratory of Bioinformatics, HUN-REN Biological Research Centre, Szeged, Hungary; ^5^ Department of Biochemistry and Medical Chemistry, Medical School, University of Pécs, Pécs, Hungary; ^6^ Macrophage Polarization Group, Institute of Genetics, HUN-REN Biological Research Centre, Szeged, Hungary; ^7^ Synthetic and Systems Biology Unit, Institute of Biochemistry, HUN-REN Biological Research Centre, Szeged, Hungary; ^8^ Department of Dermatology and Allergology, University of Szeged, Szeged, Hungary; ^9^ National Institute of Oncology, Budapest, Hungary; ^10^ Avicor Ltd., Szeged, Hungary; ^11^ Avidin Ltd., Szeged, Hungary; ^12^ Department of Physiology, Anatomy and Neuroscience, Faculty of Science and Informatics, University of Szeged, Szeged, Hungary; ^13^ CS-Smartlab Devices Ltd., Kozármisleny, Hungary

**Keywords:** tobacco smoking, stable COPD, exacerbating COPD, non-small cell lung cancer, single-cell mass cytometry, CD4 central memory T cells, CD4 effector memory T cells

## Abstract

**Introduction:**

Tobacco smoking generates airway inflammation in chronic obstructive pulmonary disease (COPD), and its involvement in the development of lung cancer is still among the leading causes of early death. Therefore, we aimed to have a better understanding of the disbalance in immunoregulation in chronic inflammatory conditions in smoker subjects with stable COPD (stCOPD), exacerbating COPD (exCOPD), or non-small cell lung cancer (NSCLC).

**Methods:**

Smoker controls without chronic illness were recruited as controls. Through extensive mapping of single cells, surface receptor quantification was achieved by single-cell mass cytometry (CyTOF) with 29 antibodies. The CyTOF characterized 14 main immune subsets such as CD4+, CD8+, CD4+/CD8+, CD4−/CD8−, and γ/δ T cells and other subsets such as CD4+ or CD8+ NKT cells, NK cells, B cells, plasmablasts, monocytes, CD11c^dim^, mDCs, and pDCs. The CD4+ central memory (CM) T cells (CD4+/CD45RA−/CD45RO+/CD197+) and CD4+ effector memory (EM) T cells (CD4+/CD45RA−/CD45RO+/CD197−) were FACS-sorted for RNA-Seq analysis. Plasma samples were assayed by Luminex MAGPIX^®^ for the quantitative measurement of 17 soluble immuno-oncology mediators (BTLA, CD28, CD80, CD27, CD40, CD86, CTLA-4, GITR, GITRL, HVEM, ICOS, LAG-3, PD-1, PD-L1, PD-L2, TIM-3, TLR-2) in the four studied groups.

**Results:**

Our focus was on T-cell-dependent differences in COPD and NSCLC, where peripheral CD4+ central memory and CD4+ effector memory cells showed a significant reduction in exCOPD and CD4+ CM showed elevation in NSCLC. The transcriptome analysis delineated a perfect correlation of differentially expressed genes between exacerbating COPD and NSCLC-derived peripheral CD4+ CM or CD4+ EM cells. The measurement of 17 immuno-oncology soluble mediators revealed a disease-associated phenotype in the peripheral blood of stCOPD, exCOPD, and NSCLC patients.

**Discussion:**

The applied single-cell mass cytometry, the whole transcriptome profiling of peripheral CD4+ memory cells, and the quantification of 17 plasma mediators provided complex data that may contribute to the understanding of the disbalance in immune homeostasis generated or sustained by tobacco smoking in COPD and NSCLC.

## 1 Introduction

Tobacco smoking frequently causes airway inflammation and oxidative stress linking the pathogenesis of chronic obstructive pulmonary disease (COPD) and lung cancer. In 2019, 212.3 million COPD cases were reported with 3.3 million deaths (1). A WHO survey showed that COPD was the third leading cause of death worldwide in 2019 (2). COPD affects more than 50% of the smoker’s population, and patients with COPD have a two-fold risk of developing lung cancer (3, 4). Previously, our group and others reviewed the molecular players and chronic inflammatory conditions in COPD establishing a lung cancer-prone microenvironment (5, 6). Free radicals such as reactive nitrogen or reactive oxygen species damage the DNA; additionally, the tobacco smoke-induced pulmonary inflammatory milieu and the genetic and epigenetic predisposition increase the mutational burden leading to the development of lung cancer (4, 5). Neoantigens were identified in COPD using mass spectrometry supporting the epidemiology data that COPD has been considered as a prestage of lung cancer (7). Non-small cell lung cancer, the most frequent comorbidity, accounts for 40%–70% of COPD patients (8). It was reported that nearly 87% of lung cancer-related deaths were affected by tobacco smoking in the USA (9). Therefore, the updated US Preventive Services Task Force (USPSTF) guidelines suggest regular diagnostic screening of patients at a minimum age of 50 with a minimum of 20 packs per year of smoking history (10). Lung cancer accounts for almost 23% of all cancer-related mortality and is one of the leading causes of cancer-related deaths with nearly 1.6 million annual deaths worldwide (11, 12).

Our current study aims to dissect the effect of tobacco smoke on the immunophenotype of the peripheral immune system in stable COPD, exacerbating COPD, and NSCLC patients. The current (2023) guideline of the Global Initiative for Chronic Obstructive Lung Disease (GOLD) defines COPD as follows: “heterogeneous lung condition characterized by chronic respiratory symptoms (dyspnea, cough, expectoration and/or exacerbations) due to abnormalities of the airways (bronchitis, bronchiolitis) and/or alveoli (emphysema) that cause persistent, often progressive, airflow obstruction” (13). COPD is a heterogeneous disease that represents a spectrum of disease severity in the range from a stable state to severe exacerbations. Stable COPD is characterized “when symptoms are well managed and pulmonary decline is minimized” (14). The exacerbation of COPD is also redefined by the recent GOLD 2023 guideline as “an event characterized by dyspnea and/or cough and sputum that worsen over ≤14 days, which may be accompanied by tachypnea and/or tachycardia and is often associated with increased local and systemic inflammation caused by airway infection, pollution, or other insult to the airways” (13). The dissection of airway inflammation in COPD is beyond the scope of our current study, but it has been reviewed elsewhere (5, 15, 16).

It is widely known that airway inflammation in COPD is associated with systemic inflammation frequently causing, e.g., cardiovascular disease (17), but the systematic investigation of the peripheral immune system in COPD has not been resolved yet completely. The markers of COPD from the biological fluids such as profiling the peripheral immune system or measuring soluble markers have been the subject of intensive research in the last years (18). Xiong et al. published recently the immunophenotyping of stable COPD and exacerbating COPD patients using an eight-member antibody panel for flow cytometry (FACS) (19). Xiong et al. found that Th1 cells, Th17 cells, Treg cell ratio, Th1/Th2 cell ratio, and the levels of C-reactive protein, interleukin (IL)-6, and IL-10 were significantly increased in patients with exCOPD. Halper-Stromberg et al. found that subjects with COPD had significantly lower levels of aggregated lymphocytes, aggregated T cells, CD4+ resting memory cells, and naive B cells and increased levels of monocytes compared with smoker controls (20). Using single-cell mass cytometry, Vasudevan et al. studied the bronchoalveolar lavage in COPD patients lacking information on the peripheral immune composition (21). Kapellos et al. used single-cell mass cytometry for the immunophenotyping of COPD patient-derived blood, but their study focused on the role of neutrophils in early-stage COPD with limited data on other compartments (22). Therefore, our mass cytometry panel was designed for the dissection of the complexity of peripheral immunity in stCOPD, exCOPD, and NSCLC.

In our study, NSCLC patients were recruited to reveal disturbances of the peripheral immune homeostasis in the context of stable or exacerbating COPD, all cases being affected by tobacco smoking, including smoker healthy volunteers. Molecular profiling and integration of multi-omics data prospectively is a novel approach to stratify patients with clinically heterogeneous diseases. High-resolution measurement characterizing a large number of cellular features was our focus in studying COPD in stable conditions vs. exacerbations. Our question of interest was the understanding of the disbalance in immunoregulation in chronic inflammatory conditions of smoker COPD cases and smoking-associated NSCLC. In our recent work, with extensive mapping of single cells, surface receptor quantification was achieved by cytometry by time-of-flight (CyTOF) with a panel consisting of 29 antibodies. Our aim was the deep immunophenotyping of COPD and NSCLC patients with a special focus on the T-cell compartment in order to shed light on the stCOPD, exCOPD, and NSCLC-associated T-cell phenotypes and the subsets of T-cell diversity. Our antibody panel consisted of markers also for the identification of other subsets such as NK cells, CD4+ NKT, CD8+ NKT, B cells, plasmablasts, monocytes, CD11c^dim^ cells, monocytoid dendritic cells (mDCs), and plasmacytoid dendritic cells (pDCs). The dissection of the complex immunophenotype may contribute to the understanding of the involvement of the immune system in the chronic inflammatory condition caused by tobacco smoking with a special focus on smoking-associated diseases such as COPD and NSCLC.

In our current study, 14 main peripheral immune cell populations were identified and characterized by single-cell mass cytometry. The CyTOF results shed light on the CD4 effector memory and CD4 central memory T cells; therefore, the whole transcriptome profiling was carried out for these FACS-sorted particular cell types in all smoking conditions, such as healthy controls, stable COPD, exacerbating COPD, and NSCLC patient-derived samples. The concentration of 17 soluble mediators was also measured by the Luminex MAGPIX^®^ system from the plasma samples to dissect the pattern of relevant cytokines/chemokines and immune checkpoint modulators circulating in the peripheral blood.

## 2 Materials and methods

### 2.1 Study subjects

The following subjects were recruited: 1) smoker healthy controls (SmHC) without chronic illness and without regular medication. 2) Stable COPD patients (stCOPD) were selected according to the guideline of GOLD with a smoking history and without known lung cancer. 3) Acute exacerbating COPD patients (exCOPD) were also selected based on the GOLD guideline; the withdrawal of the blood was before the glucocorticoid therapy. ExCOPD patients were selected with smoking history and without known lung cancer. ExCOPD was defined as increased dyspnea, cough, or sputum expectoration (quality or quantity) that led the subject to seek medical attention, as specified in international guidelines (23). 4) Smoker, non-small cell lung cancer (NSCL) patients were involved before receiving therapy. Bacterial or viral infection during the exacerbation was not tested. Samples were collected from December 2018 to December 2019 before the outbreak of COVID-19. All subjects were over 18 and self-conscious. The subjects gave their informed consent before participating in the study. The study was conducted in accordance with the Declaration of Helsinki, and the protocol (“Immunophenotyping in COPD and lung cancer”) was approved by the Ethics Committee of the National Public Health Center under the 33815-7/2018/EÜIG Project identification code.

### 2.2 Study design

Subjects were recruited for CyTOF from the following groups: 1) smoker healthy control (without known disease and without regular medication, *n* = 9), 2) smoker-stable COPD (*n* = 8) patients, 3) smoker-exacerbating COPD patients before receiving corticosteroid treatment (*n* = 8), and 4) therapy-naive NSCLC patients without chemotherapy and/or immunotherapy (*n* = 8). Subjects were recruited for Luminex from the following groups: 1) smoker healthy control (*n* = 9), 2) smoker-stable COPD (*n* = 11) patients, 3) smoker-exacerbating COPD patients before receiving corticosteroid treatment (*n* = 13), and 4) therapy-naive NSCLC patients without chemotherapy and/or immunotherapy (*n* = 13), This is a cross-sectional study with the collection of 20 mL venous peripheral blood at one time point (before receiving medication where it was applicable). Demographic and clinical data of the patients are summarized in **Supplementary Table 1**.

### 2.3 PBMC isolation

After the collection of 20 mL blood into an EDTA vacutainer (Becton Dickinson, Franklin Lakes, USA), PBMCs were purified by Leucosep tubes (Greiner Bio-One, Kremsmünster, Austria) according to the manufacturer’s instructions. If the pellet was light red, 2 mL of ACK lysing buffer (ACK: 0.15 M of NH_4_Cl, 10 mM of KHCO_3_, 0.1 mM of Na_2_EDTA, pH 7.3; Merck, Darmstadt, Germany) was applied at room temperature (RT) for 2 min. Samples were washed twice with 10 mL of PBS (Merck) and subsequently cell counted, and viability check was performed with Trypan Blue exclusion. Cryopreservation of PBMCs was carried out in stocks of 4 × 10^6^ cells of 1 mL of FBS (Capricorn Scientific, Ebsdorfergrund, Germany) supplemented with 1:10 of DMSO (Merk) [v/v] in cryotubes (Greiner Bio-One) in liquid nitrogen (Messer, Bad Soden, Germany).

### 2.4 Cell preparation for CyTOF

Cells were processed for CyTOF as described previously by our group (24). Briefly, cryotubes were thawed in a 37°C water bath for 2 min, and cells were transferred into a 14-mL 37°C warm RPMI (Capricorn Scientific) and centrifuged at 350×*g* for 6 min at RT. PBMCs were washed again with 5 mL of Maxpar Staining Buffer (MCSB, Fluidigm, South San Francisco, CA, USA) and centrifuged at 350×*g* for 6 min at RT, cells were counted in 1 mL of PBS, and viability was determined with Trypan Blue exclusion.

### 2.5 Antibody staining

The antibody staining of cells for CyTOF was performed as described previously by our group with minor modifications (25, 26). Briefly, viability was determined by cisplatin (5 µM of ^195^Pt, Fluidigm) staining for 3 min on ice in 300 µL of RPMI. The sample was diluted by 2 mL of RPMI (MCSB, Fluidigm) and centrifuged at 350×*g* for 5 min. PBMCs were washed again with 4 mL of MCSB and centrifuged at 350×*g* for 6 min at RT. Cells were resuspended in 50 µL of MCSB supplemented with 1:20 v/v TrueStain FcX™ FC receptor blocking solution (BioLegend, San Diego, USA) and incubated at RT for 10 min. Cells were stained with 29 antibodies of the Human Immune Monitoring Panel (Fluidigm) and incubated at 4°C for 45 min. The antibodies used for CyTOF are listed in **Supplementary Table 2**. Cells were washed twice with 1 mL of MCSB and prefixed with 1 mL of Pierce™ formaldehyde (Thermo Fisher Scientific, Waltham, MA, USA) solution diluted in PBS to 1.6% and incubated at RT for 10 min. Stained and prefixed cells were centrifuged at 800×*g* for 6 min at RT and resuspended in 1 mL of Fix & Perm solution (Fluidigm) supplemented with 1:1,000 v/v Ir^191^/Ir^193^ DNA intercalator (Fluidigm) for overnight incubation.

### 2.6 CyTOF data acquisition and data preprocessing

The acquisition of the samples for CyTOF was executed as described previously by our group with minor modifications (27, 28). Briefly, samples were washed three times with MCSB before being filtered through a 30-μm Celltrics (Sysmex Hungaria Kft, Hungary) gravity filter, and the cell concentration was adjusted to 7 × 10^5^/mL in Maxpar Cell Acquisition Solution (Fluidigm). Finally, EQ four-element calibration beads (Fluidigm) were added at a 1:10 ratio (v/v) and acquired on a properly tuned Helios mass cytometer (CyTOF, Fluidigm), and 1 × 10^6^ events per individual PBMC were collected to be able to identify rare cell subsets. The generated flow cytometry standard (FCS) files were randomized and normalized with the default setting of the internal FCS processing unit of the CyTOF software (Fluidigm, version 7.0.8493).

### 2.7 CyTOF data processing

**Figure 1:**
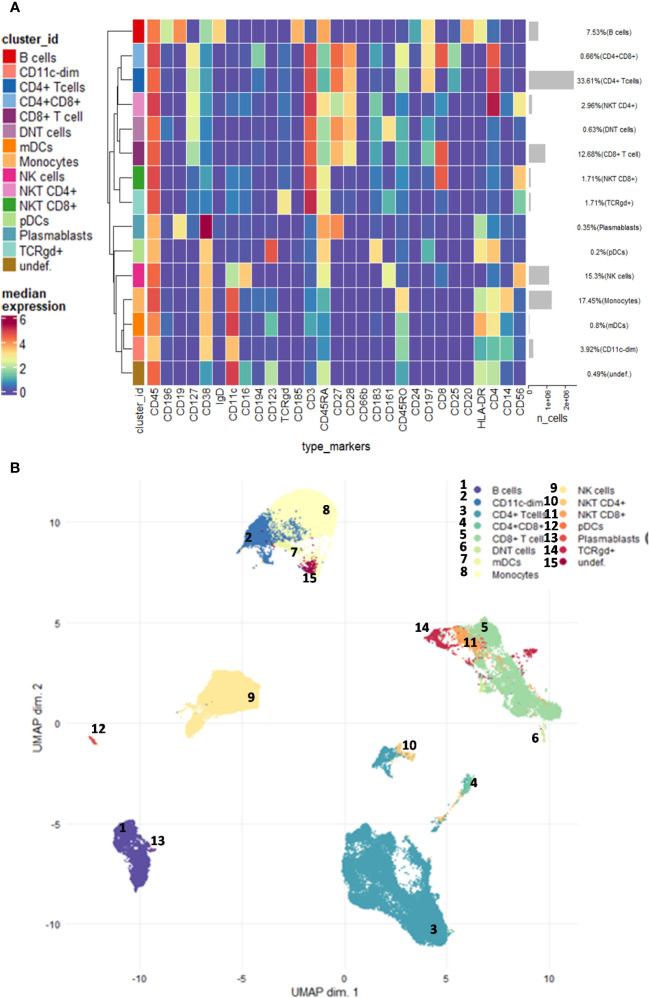
Demonstration of the studied main immune subsets. **(A)** Unsupervised clustering and the heatmap of expression intensities of the analysed immune cells. **(B)** The FlowSOM (Self-Organizing Maps for flow cytometry) and UMAP (Uniform Manifold Approximation and Projection for Dimension Reduction) analysis identified 14 main subsets as follows: CD4+ T cells, CD8+ T cells, CD4+CD8+ T cells, DN (double-negative) T cells (CD4−CD8−), γ/δ T cells, NK cells, CD4+ NKT cells, CD8+ NKT cells, plasmablasts, B cells, monocytes, CD11c^dim^ cells, plasmacytoid dendritic cells (pDCs), and monocytoid dendritic cells (mDCs). The analysis was carried out on the entire dataset in the R software including 33 FCS files.

Randomized and normalized FCS files were uploaded to the Cytobank analysis platform (Beckman Coulter, Brea, USA). Exclusion of normalization beads, dead cells, debris, and doublets was performed. There were no significant differences in cell counts between the examined groups. The FCS files with the CD45-positive living singlets were exported and further analyzed in R. Compensation methodology, FlowSOM clustering, and reduction of dimensionality were adapted from Crowell HL et al. [BioConductor CATALYST, compensation (29): data analysis (30):]. Utilizing the CATALYST and flowCore R package, FCS files were integrated, compensated, and transformed. After signal spillover compensation, CyTOF marker intensities were inverse hyperbolic sine-transformed (arcsinh) with cofactor 5. For the main population definition, we carried out a Self-Organizing Maps-based method (FlowSOM) metaclustering on compensated and transformed files. We identified different metaclusters which were separately subclustered with another round of FlowSOM. High-dimensional reduction and visualization were performed using the t-distributed stochastic neighbor embedding (t-SNE) algorithm/method. A total of 300,000 cells and 29 markers were used to create the peripheral human immune system UMAP (Uniform Manifold Approximation and Projection for Dimension Reduction) (**Figure 1**).

### 2.8 RNA sequencing

Cell sorting of CD4+ EM or CD4+ CM T cells was carried out. The PBMCs were thawed from liquid nitrogen. Cells were counted in PBS, and viability was determined with Trypan Blue exclusion. Cells were resuspended in 50 µL of PBS supplemented with 1:20 v/v TrueStain FcX™ FC receptor blocking solution (BioLegend) and incubated at RT for 10 min. The enrichment of CD4+ T cells was performed using the Dynabeads™ FlowComp™ Human CD4 Isolation Kit from PBMC following the instruction of the manufacturer (Thermo Fisher Scientific). After titrating the antibodies, the CD4+ T cells were incubated with CD45RA-FITC (Hl100 clone), CD45RO PERC/Cy5.5 (UCHL1 clone), and CD197(CCR7)-PB (G043H7 clone) (BioLegend) 25× diluted in FACS buffer (2% FBS in PBS) in 50 µL at RT for 45 min. After washing with 1 mL of FACS buffer and centrifugation at 350×*g* for 5 min, cells were resuspended in FACS buffer and 2 mM of EDTA for cell sorting using FACSJazz (Beckman Coulter). Manual gating was used to sort 1) CD4+ EM (CD45RA−/CD45RO+, CD197−) or 2) CD4+ CM (CD45RA−/CD45RO+, CD197+) cells. Cells were centrifuged at 350×*g* for 5 min and resuspended in 500 µL of TRIzol (Thermo Fisher Scientific) and stored at −80°C. The isolation of RNA was performed using the RNeasy Micro kit (Qiagen, Hilden, Germany). The RNAse inhibitor was added 20 U/µL diluted 100× (Thermo Fisher Scientific). The concentration and purity of RNA was measured using NanoDrop™ One (Thermo Fisher Scientific). Because of the relatively low number of memory cells and yielded RNA from one subject, the RNA samples of one study cohort were pooled in an equivalent manner (the same amount from three to five subjects within one group). The RNA sequencing (Massive Analysis of cDNA Ends = MACE = 3′ mRNA-Seq) was performed by GenXPro GMBH (Frankfurt Am Main, Germany) as a service. The normalized gene expression data of the RNA-Seq are listed in **Supplementary Table 3**.

### 2.9 Gene set enrichment analysis

Gene set enrichment analysis was performed using GSEA 4.1.0 (31). We ran the software in CLI mode and analyzed its outputs through custom R scripts, attached as a supplementary R Markdown file (**Supplementary File 1**).

### 2.10 Measurement of plasma proteins

The measurement of plasma proteins was performed as described previously by our group with minor modifications (32–34). Briefly, after the withdrawal of 20 mL of blood into an EDTA vacutainer (Becton Dickinson), human peripheral blood mononuclear cells and plasma samples were purified by Leucosep tubes (Greiner Bio-One, Austria). Plasma fractions were stored at −80°C in aliquots before running the assay. Luminex xMAP (MAGPIX®) technology was used to determine the protein concentrations of 17 distinct immuno-oncology checkpoint proteins (BTLA, CD28, CD80, CD27, CD40, CD86, CTLA-4, GITR, GITRL, HVEM, ICOS, LAG-3, PD-1, PD-L1, PD-L2, TIM-3, TLR-2) performing the Human Immuno-Oncology Checkpoint Protein Panel 1 - Immuno-Oncology Multiplex Assay (Cat. num.: HCKP1-11, Merck) according to the instructions of the manufacturer. Briefly, all samples were thawed and tested in a blind fashion. A 50-μl volume of each sample, 25 μl of standard, 25 μl of matrix solution, and 25 μl of universal assay buffer were added to a 96-well plate (provided with the kit) containing 25 μl of capture antibody-coated, fluorescent-coded beads. A biotinylated detection antibody mixture and streptavidin-PE were added to the plate after the appropriate incubation period. After the last washing step, 150 μl of drive fluid was added to the wells, and the plate was incubated for an additional 5 min and read on the Luminex MAGPIX® instrument. Luminex xPonent 4.2 software was used for data acquisition. Five-PL regression curves were generated to plot the standard curves for all analytes by the Analyst 5.1 (Merck) software calculating with bead median fluorescence intensity values. The panel of the investigated 17 plasma proteins and the range of detection (in pg/mL from the lower limit to the upper limit) are available in **Supplementary Table 4**. Data were pooled from two independent measurements and plotted in GraphPad Prism v8 (Dotmatics, Boston, USA).

### 2.11 Clustering of samples based on cytokine levels

The concentration levels of analytes, measured in samples from four patient categories (smoker healthy control, stable COPD, exacerbated COPD, treatment-naive smoker NSCLC) were visualized on a heatmap using the pheatmap R package (35). Hierarchical clustering was carried out based on Euclidian distance and using the hclust method in R.

### 2.12 Statistical analysis

In CyTOF, median signal intensities, cell frequencies, and subpopulation frequencies were analyzed with GraphPad Prism 8.0.1. The normality of distributions was tested with D’Agostino & Pearson test and passed if all the group’s alpha values were under 0.05. Normally distributed datasets were compared with the ordinary one-way ANOVA or Brown-Forsythe ANOVA when the standard deviations were not equal. For non-parametric analysis, the Kruskal–Wallis test was applied. All types of significance tests were corrected for multiple comparisons by controlling the false discovery rate (FDR) with the two-stage Benjamini, Krieger, and Yekutieli approach with an FDR cutoff of 10%. Differences are considered significant at *p < 0.05, **p < 0.01, and ***p < 0.001 (error bars specify means ± SD). In Luminex, statistical significance was calculated using Student’s *t*-test. Statistical computations were done by dplyr v1.0.6 and rstatix v0.7.0, while graph generation was done by ggpubr v0.4.0 and ggprism v1.0.3 R packages.

## 3 Results

### 3.1 Single-cell immunophenotyping of peripheral mononuclear cells shed light on the disbalance of immune homeostasis in stable COPD, exacerbating COPD, and NSCLC

Using single-cell mass cytometry with the 29-membered antibody panel of the commercially available Human Immune Monitoring kit, we could identify the main immune subsets in the peripheral blood of the studied groups, such as smoker healthy controls (SmHCs), smoker-stable COPD (stCOPD) patients, smoker-exacerbating COPD (exCOPD) patients, and smoker NSCLC patients. During the data analysis, calibration beads were excluded, and singlets, live cells, and CD45+ cells were manually gated. Further unsupervised analysis was performed on CD45+ living singlets where the following populations were excluded by manual gating: CD3+CD14+, CD3+CD19+, CD19+CD14+, and CD19+CD56+ (**Supplementary Figure 1**) The FlowSOM (Self-Organizing Maps for flow cytometry) and UMAP analysis identified 14 main subsets, namely, CD4+ T cells, CD8+ T cells, CD4+CD8+ T cells, DN (double-negative) T cells (CD4−CD8−), γ/δ T cells, NK cells, CD4+ NKT cells, CD8+ NKT cells, plasmablasts, B cells, monocytes, CD11c^dim^ cells, pDCs, and mDCs (**Figure 1**; **Supplementary Figure 2**). The frequency of the main subsets was quantified and expressed as the % of living CD45 singlets (**Supplementary Figure 2**). The CD4+ T cells were reduced in stCOPD and exCOPD to one-third and half of the SmHCs, respectively (**Supplementary Figure 2A**). The percentage of CD8+ T cells outperformed the SmHCs, but in the exCOPD, these cells were also half of the SmHCs (**Supplementary Figure 2A**). The DN T cells were also the lowest in exCOPD. Conversely, the percentage of monocytes was two times higher in exCOPD than in SmHCs; the CD11c^dim^ cells were 10 times higher both in stCOPD and exCOPD compared with the SmHCs and five times higher than in the NSCLC group. Both the pDCs and mDCs were with the lowest frequency in the exCODP group (**Supplementary Figure 2D**).

**Figure 2:**
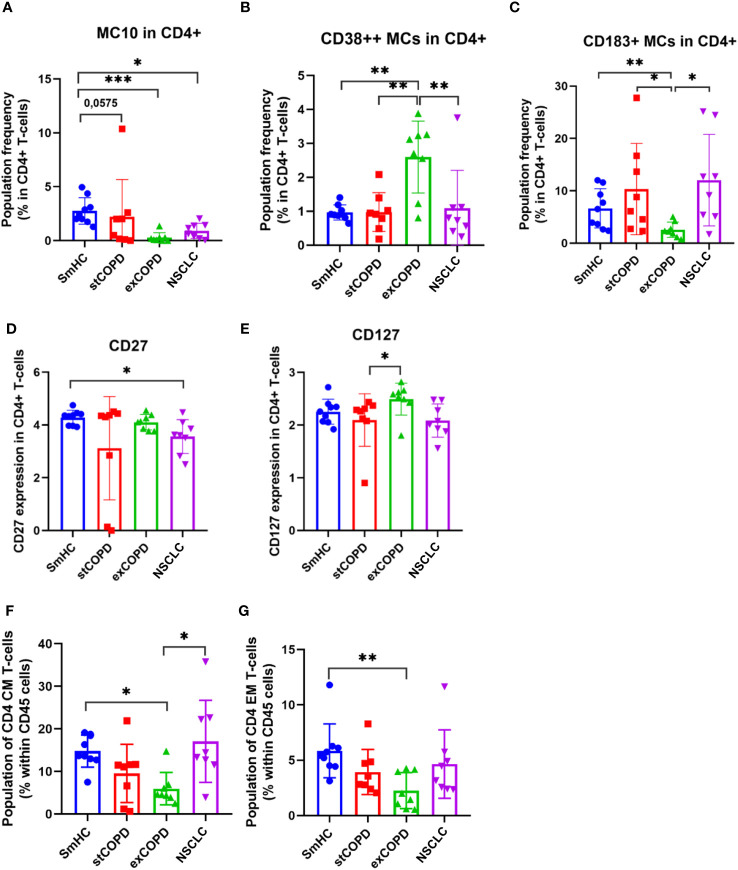
The population frequency **(A–C)** and marker expression profile of CD4+ T cells **(D, E)**. The differences in the percentage of CD4+ CM **(F)** and CD4+ EM cells **(G)**. *p < 0.05, **p < 0.01, ***p < 0.001.

The activation of adaptive immunity in smoker COPD patients and the dysfunction/exhaustion of T cells in lung cancer have long been known (36–38). Therefore, our focus was on the analysis of T-cell subsets. Fourteen metaclusters (MCs) were identified in the CD4+ T cells (**Supplementary Figure 3**). Significant differences were found in MC10 (CD4+/CD185+) with the decrease in exCOPD and NSCLC (**Figure 2A**); the merged MC13 and MC14 were the CD38^bright^ CD4+ T cells (CD38^++^ MCs) with the highest frequency in exCOPD (**Figure 2B**); conversely, the CD183+ MCs (MC5+MC6+MC8+MC10) were the lowest in exCOPD (**Figure 2C**). Regarding marker expressions on all CD4+ T cells, CD27 decreased in NSCLC vs. SmHC (**Figure 2D**), and CD127 decreased in stCOPD vs. exCOPD (**Figure 2E**). Our interest turned toward CD4 memory cells, such as CD4+ CM (central memory) cells and CD4 EM (effector memory). The CD4+ CM cells were defined as CD4+/CD45RA−/CD45RO+/CD197+ merging 03, 10, 11, and 13 MCs. The CD4+ EM cells were defined as CD4+/CD45RA−/CD45RO+/CD197− merging 02, 04, 09, and 14 MCs. The percentage of CD4+ CM and CD4+ EM cells was the lowest in exCOPD (**Figures 2F, G**).

**Figure 3:**
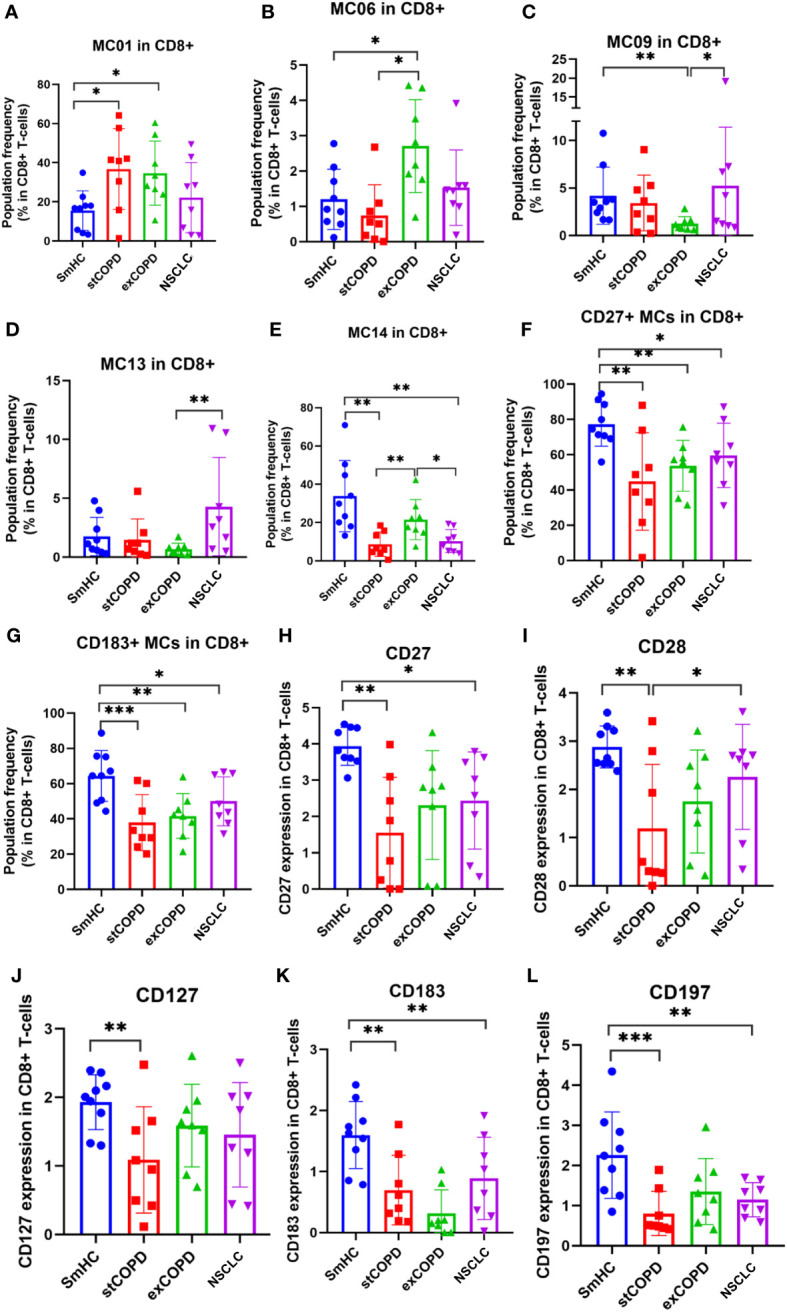
The population frequency **(A–G)** and marker expression profile of CD8+ T cells **(H–L)**. *p < 0.05, **p < 0.01, ***p < 0.001.

Next, the CD8+ T cells were clustered, and 15 MCs were defined (**Supplementary Figure 4**). Significant differences were found in the following MCs as shown in **Figure 3**: MC01: CD45RA^dim^, MC06: CD28+/CD194+, MC09: CD27+/CD45RO+, MC13: CD27+/CD183+/CD194^dim^, and MC14: CD45RA+/CD197+ (**Figure 3**). The CD27+ MCs represent cells of the MC04, MC05, MC07, MC08, MC09, MC11, MC13, MC14, and MC15, which were the lowest in stCOPD (**Figure 3F**). The CD183+ MCs represent cells of the MC04, MC07, MC09, MC11, MC12, MC13, and MC14, which were also the lowest in stCOPD (**Figure 3G**). The expression of CD27 and CD28 showed a similar pattern with the lowest frequency in the stCOPD (**Figures 3H, I**). The CD127 was significantly reduced in stCOPD vs. SmHC (**Figure 3J**), and the CD183 and CD197 were reduced in stCOPD and NSCLC vs. SmHC (**Figures 3K, L**).

**Figure 4:**
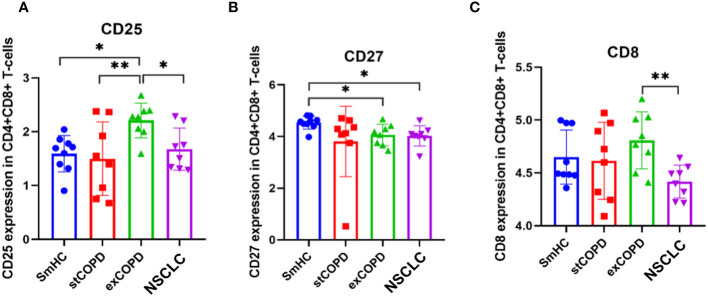
Marker expression **(A)** CD25, **(B)** CD27, **(C)** CD8 profile of CD4+CD8+ T-cells. *p* *<0.05, **<0.01.

Analyzing the CD4+CD8+ double-positive T cells, CD25 was elevated in exCOPD, CD27 was decreased in exCOPD and NSCLC, and CD8 was the lowest in NSCLC (**Figures 4A–C**).

**Figure 5:**
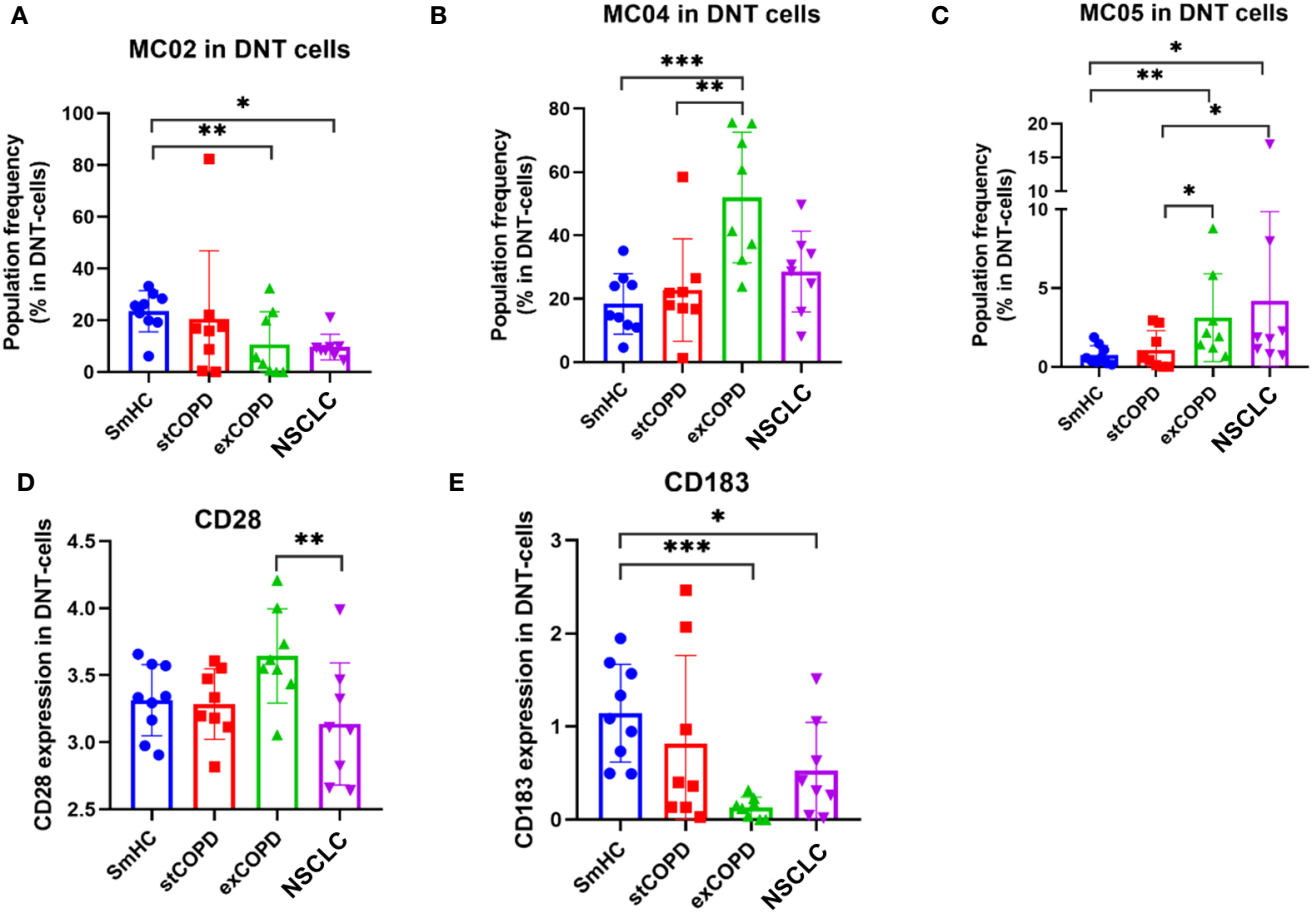
The population frequency of the metaclusters in CD4−CD8− (DN) T cells **(A–C)** and the marker expression profile of **(D)** CD28 and **(E)** CD183 on the surface of DN T cells. *p < 0.05, **p < 0.01, ***p < 0.001.

The double-negative CD4−CD8− (DN) T cells delineated five MCs (**Supplementary Figure 5**). Significant differences in the frequency of the cells were recorded in the following MCs: MC02: CD27+/CD127+/CD161+ that was decreased in exCOPD and NSCLC, MC04: CD38++ that was in excess in exCOPD, and MC05: CD38++/CD183+/CD196+ that was gradually increased from stCOPD to exCOPD and further increased in NSCLC (**Figures 5A–C**). The surface expression of CD28 decreased in NSCLC vs. exCOPD, and CD183 expression was lower in exCOPD and in the NSCLC groups vs. SmHC (**Figures 5D, E**).

**Figure 6:**
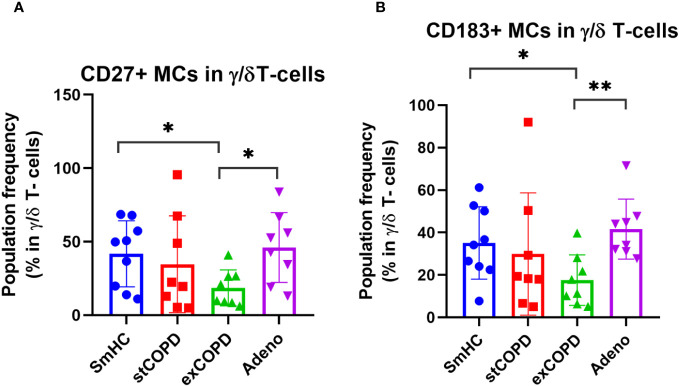
The population frequency of **(A)** CD27+ or **(B)** CD183+ MCs in γ/δ T-cells. *p* *<0.05, **<0.01.

The γ/δ T cells were divided into 11 MCs (**Supplementary Figure 6**). The CD27+ γ/δ T cells were determined merging MC03, MC04, MC05, MC06, MC07, and MC09 MCs. The CD27 expression on the surface γ/δ T cells was decreased in exCOPD compared with SmHC and NSCLC (**Figure 6A**). The CD27+ γ/δ T cells were determined merging MC03, MC04, MC06, MC07, and MC11 MCs. The CD183 expression in the γ/δ T-cell subset decreased in stCOPD and exCOPD compared with SmHC (**Figure 6B**).

The deep immunophenotyping using CyTOF revealed significant differences in 46 specific markers/populations of other, non-T-cell compartments, but because of the focus of our current study on T cells, the other subtypes are listed in the **Supplementary Material**, such as CD4+ NKT cells and CD8+ NKT cells (**Supplementary Figure 7**), classic NK cells (**Supplementary Figures 8**, **9**), B cells (**Supplementary Figures 10**, **11**), plasmablasts (**Supplementary Figure 12**), monocytes (**Supplementary Figures 13**, **14**), CD11c^dim^ cells, and mDCs (**Supplementary Figures 15**, **16**). However, it is worth emphasizing some of the significant differences which may be relevant in the context of exacerbation of inflammation in the impairment of the antitumor immune response, such as the CD16 (FcγRIII) decrease both in CD4+ NKT and CD8+ NKT cells in NSCLC compared with SmHC (**Supplementary Figure 7**). We could detect the increase of the G-protein-coupled receptors, such as the CD183 (CXCR3) or CD194 (CCR4) on the cell surface of NK cells in NSCLC compared with the other three groups (**Supplementary Figure 9**). The MC08 metacluster of B cells positive for CD19+/CD20+/CD45RA+/CD185+/CD27+ was dramatically decreased in stCOPD, exCOPD, and NSCLC compared with SmHC (**Supplementary Figures 10**, **11**). The expression of CD27, CD19, CD185 (CXCR5), and CD196 (CCR6) was reduced on the surface of B cells in the NSCLC group (**Supplementary Figure 11**). CD19, CD27, and CD45RA were also decreased on the surface of plasmablasts (**Supplementary Figure 12**). The main significant changes in the myeloid compartment of the PBMCs are also highlighted. The cells of the MC03 (CD14+/CD11c^high^+/CD38+/CD45RO+/HLA-DR^low^+), the classical monocytes (CD14+/CD16−), and HLA-DR^low^ cells were significantly higher in exCOPD among monocytes (**Supplementary Figures 13**, **14**). On the contrary, MC06 (CD14+/CD11c^high^/CD38+^low^/CD123^dim^/CD45RO+/HLA-DR^high^+), MC07 (CD14+/CD11c^high^/CD38+/CD45RO+/HLA-DR+), MC08 (CD14+/CD11c^high^/CD38+/CD45RO^high^, HLA-DR^low^+), and CD16+ cells were the lowest in exCOPD in the monocyte compartment (**Supplementary Figures 13**, **14**). The MC01 (CD196^high^) of CD11c^dim^ cells was the lowest in exCOPD, but the MC03 (CD196−) cells were of the highest frequency in exCOPD among CD11c^dim^ cells (**Supplementary Figures 15**, **16**).

### 3.2 The transcriptome of CD4+ EM or CD4+ CM cells showed a correlation in NSCLC with exacerbating COPD samples

**Figure 7:**
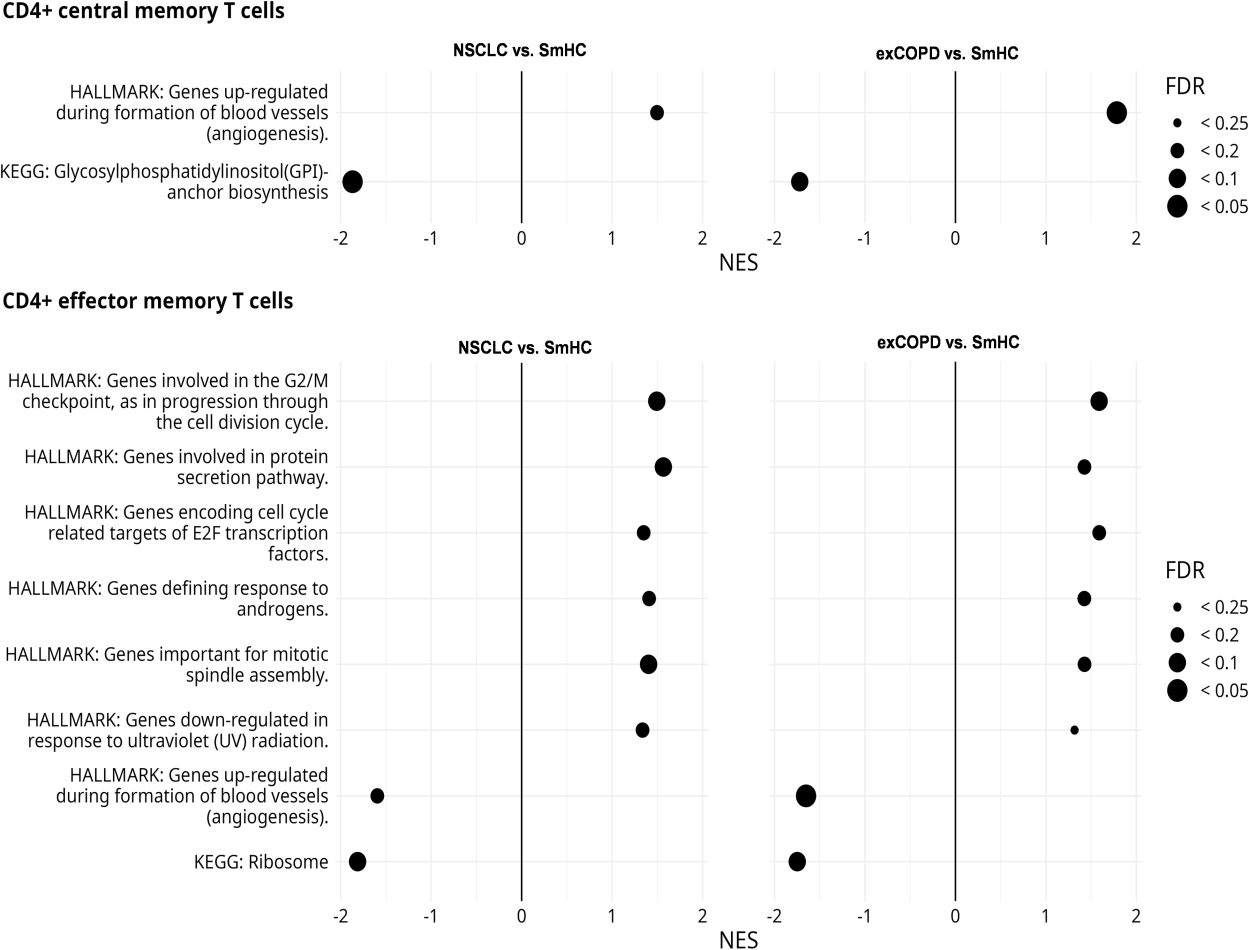
Results of gene set enrichment analyses of CD4+ central memory and effector memory T cells. The figure shows those Hallmark and KEGG gene sets that had similar trends in NSCLC vs. smoker healthy controls (SmHCs) and exacerbated COPD vs. SmHC comparisons. Positive normalized effect score (NES) values indicate enrichment in control. Point sizes indicate false discovery rate (FDR) value ranges.

The CD183 (CXCR3) showed reduced expression in the aforementioned T-cell subsets, and its influence on the development of memory T cells is known (39). Additionally, alterations in memory T-cells subsets were reported in COPD previously (40–42). Therefore, the CD4+ EM and CD4+ CM cells were FACS-sorted for transcriptomic analysis (RNA sequencing) in our study. The purified RNA was pooled from three to five subjects within one study cohort in an RNA equivalent manner in order to avoid bias from differences in the amount of isolated RNA (**Supplementary Table 3**). The pooled RNA-Seq identified 203 differentially upregulated and 167 differentially downregulated genes in the CD4+ CM cells of exacerbating COPD and NSCLC samples showing 0.807 Pearson correlation (**Supplementary Figure 17**). The pooled RNA-Seq identified 234 differentially upregulated and 90 differentially downregulated genes in the CD4+ EM cells of exacerbating COPD and NSCLC samples showing 0.747 Pearson correlation (**Supplementary Figure 18**). The normalized expression data of the full list of the results of RNA-Seq are found in **Supplementary Table 3**. The gene set enrichment analyses (GSEA) showed a similar pattern between lung NSCLC and exacerbating COPD-derived peripheral CD4+ CM and CD4+ EM cells (**Figure 7**). The Hallmark and Kyoto Encyclopedia of Genes and Genomes (KEGG) gene sets were analyzed for the normalized expression values in relation to the healthy control samples. Pathways in angiogenesis were upregulated in CD4+ CM cells both in exCOPD and NSCLC, and pathways in cell division, protein secretion, transcription, response to androgens, or response to ultraviolet radiation were upregulated in CD4+ EM cells both in exCOPD and NSCLC. The 10 most repressed genes were in CD4+ CM cells both in exCOPD and NSCLC samples: MAP3K2, SUZ12, SYNE2, HNRNPF, RNF24, APLP2, HIPK3, FBXL5, SEPT2, and GLCCI1. The 10 most upregulated genes were in CD4+ CM cells both in exCOPD and NSCLC samples: AC008481.1, COMMD1, MRPL41, C12orf57, RPS3AP5, AL009174.1, AC026979.2, AC108161.1, CUTA, and AL450998.1. The 10 most repressed genes were in CD4+ EM cells both in exCOPD and NSCLC samples: FGFBP2, CERKL, RBL2, GNLY, SYNE1, HNRNPLL, PTPN4, GLS, ITGAM, and TGFBR3. The 10 most upregulated genes were in CD4+ EM cells both in exCOPD and NSCLC samples: AC008677.2, AC245033.4, AL450998.1, RPS18P9, RPS3AP5, EEF1A1P5, RPL27AP, RPL41P5, AC008481.1, and S100A9. The gene ID, long name of the genes, and normalized expression values are listed in **Supplementary Table 3**.

### 3.3 The clustering based on the concentration of the immuno-oncology checkpoint soluble mediators in the plasma of the peripheral blood

**Figure 8:**
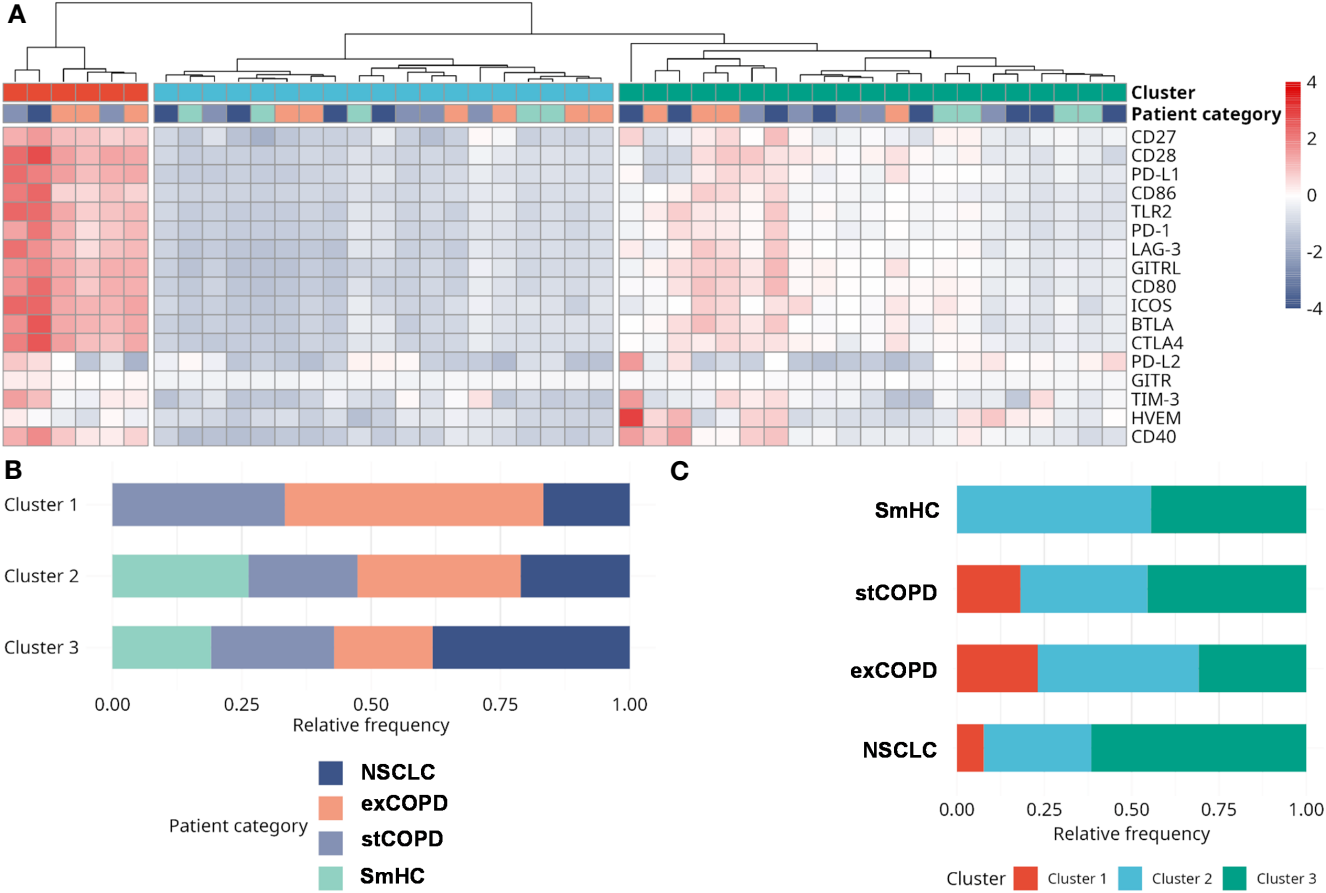
The analysis of the concentration of immuno-oncology mediators of the plasma of the patients. **(A)** Analyte concentrations in different samples are shown color-coded. Concentration levels have been normalized using the Z-score transformation. Three main sample clusters were identified using hierarchical clustering. **(B)** The distribution of patient categories in the three clusters. **(C)** The distribution of clusters among different patient categories. Clusters 1 and 2 can be characterized by the highest and lowest analyte levels, respectively. Cluster 3 shows intermediate analyte levels and contains the largest fraction of NSCLC samples.

After the withdrawal of peripheral blood, the PBMCs were purified for CyTOF and RNA-Seq analysis, but the plasma of the peripheral blood was used for the quantitative analysis of immuno-oncology mediators using the multiplex Luminex MAGPIX® technology. The list of the analyzed soluble proteins, the UniProt ID, and assay sensitivity are listed in **Supplementary Table 4**. The individual concentration values of the 17 measured proteins and multiple comparisons among the four studied groups are visualized on the scatter plots in **Supplementary Figure 19**. We highlighted only the significant differences in the text. The inhibitory CD366 (TIM-3) was elevated in stCOPD vs. SmHC, and it was lower in exCOPD vs. stCOPD. The inhibitory CD273 (PD-L2) was significantly the lowest in exCOPD compared with the other three groups. The T-cell stimulatory CD86 (B7-2) and GITRL were higher in exCOPD vs. SmHC (**Supplementary Figure 19**). For better clarity of the results, clustering was applied and is shown in **Figure 8**. The concentration values after the Z-score transformation resulted in three main clusters (**Figure 8A**). Cluster 1 with the highest concentration of inflammatory mediators lacks SmHCs and includes three exCOPD patients, two stCOPD patients, and one NSCLC patient. Cluster 2 with the lowest concentration of the measured soluble mediators includes five SmHCs, six exCOPD, four stCOPD, and four NSCLC patients. The intermediate cluster 3 in terms of the medium expression of immuno-oncology mediators represents four SmHCs, five stCOPD, four exCOPD, and eight NSCLC patients.

## 4 Discussion

The aim of our study was to have a deeper understanding of the disbalance in the immune homeostasis of smoker-stable COPD and smoker-exacerbating COPD. Since tobacco smoking and an established COPD frequently lead to lung cancer, human smoker NSCLC samples were also investigated. The self-perpetuating systemic inflammation caused by tobacco smoking has been intensively studied earlier by our group and others (5, 43, 44). Cigarette smoking caused alterations in the peripheral immune landscape in COPD that has been reviewed by Taucher et al. (45). Using single-cell mass cytometry, we have recently shown the peripheral immunophenotype of smoker advanced NSCLC patients who underwent chemotherapy or immune checkpoint inhibitor (ICI) therapy (34). However, a limited number of studies are available using CyTOF for the immunophenotyping of the PBMCs of smoker COPD and NSCLC samples. Previously, Vasudevan et al. showed a lower PD-L1 and PD-L2 expression of myeloid cells that supports chronic inflammation in the bronchoalveolar lavage (BAL) of smoker COPD patients using CyTOF (21). Freeman et al. used a 12-color FACS panel for the immunophenotyping of PBMC BAL and sputum samples of COPD cases (46). They published gating strategies to define seven populations (CD4+ and CD8+ T cells, B cells, monocytes, macrophages, neutrophils, and eosinophils) and proposed future analysis to reveal the correlation of immune subset phenotypes with smoking history, spirometry, and other physiologic parameters of the COPD patients. Using an 8-color FACS panel, Xiong et al. showed an increase of Th1, Th17, and Treg phenotypes in the peripheral blood of acute exacerbating COPD patients compared with stCOPD (19). In a murine model of cigarette smoke exposure, Kapellos et al. showed an expansion of CD11b+Ly6G+CD117−CD62L−CD172a− neutrophils in the bone marrow using CyTOF (22). According to our current knowledge, our study is the first to compare the peripheral immunophenotype of smoker healthy controls with stCOPD, exCOPD, and smoker NSCLC. The peripheral blood of the patients was extensively studied by a 29-member antibody panel for single-cell mass cytometry. We could determine and analyze 14 immune subsets, namely, CD4+ T cells, CD8+ T cells, CD4+CD8+ T cells, DN (double-negative) T cells (CD4−CD8−), γ/δ T cells, NK cells, CD4+ NKT cells, CD8+ NKT cells, plasmablasts, B cells, monocytes, CD11c^dim^ cells, pDCs, and mDCs. The extensive immunophenotyping of T cells is shown in the main text, and additionally, 46 further significant differences are shown about NK, NKT, B, or myeloid cells in the **Supplementary Material**. The ratio of CD4+ T cells and CD8+ T cells was significantly elevated in NSCLC at the expense of the reduction of monocytes and CD11c^dim^ cells. An increased expression of CD38, an activation marker functioning as a cyclic ADP ribose hydrolase, was shown on CD4+ T cells. The increased CD38-mediated signaling in COPD and the generation of adenosine diphosphate ribose and cyclic adenosine diphosphate ribose were described previously by Guedes et al. (47). The CD183 and the chemokine receptor CXCR3 may interact with CXCL9, CXCL10, and CXCL11 affecting T-cell polarization (48), and we found that CD183+ MCs were in the lowest frequency both in CD4+ T cells in exCOPD and in CD8+ T cells in stCOPD. The CD183 expression was also reduced in DN T cells in exCODP and in γ/δ T cells in stCOPD. The CD183 may affect memory T-cell development (39, 49). Indeed CD4+ CM and CD4+ EM cells were in the lowest frequency in exCOPD cases. Roberts et al. published earlier the lower percentage of CD4+ CM and CD4+ EM cells in COPD compared with smoker controls (40). Because of the central role of CD4+ T cells in the polarization of T-cell-mediated responses, the CD4+ CM and CD4+ EM cells were selected for FACS sorting and whole transcriptome analysis by RNA-Seq. This is the first study to show the peripheral immunophenotyping and transcriptome of human patient-derived CD4+ CM and CD4+ EM cells from smoker controls, stCOPD, exCOPD, and NSCLC subjects. The chronic inflammation persistent in exacerbating COPD and NSCLC showed 0.8 or 0.74 Pearson correlation of the co-expression of differentially expressed genes in CD4+ CM or CD4+ EM cells, respectively. Some of the markers such as S100A9 are induced by tobacco smoking and contribute to the development of COPD or decreased survival in NSCLC (50, 51). Neoantigens are generated during tobacco smoking both in COPD and lung cancer (7), and naive CD4+ T cells are exposed to HLA-demonstrated epitopes that may lead to the development of antigen-specific long-lived memory T cells. The CD4+ CM T cells favor trafficking in the primary or secondary lymphoid organs from the periphery, while CD4+ EM T cells migrate to the non-lymphoid sites of inflammation (52). The analysis of the tissue-resident immune composition of COPD and lung cancer has been published previously by other groups (41, 53, 54). Our focus was on the characterization of peripheral immunity, the dissection of single-cell heterogeneity of the subsets of PBMCs, and the quantitative measurement of peripheral immuno-oncology mediators. Sahin et al. published about the serum biomarkers in stCOPD and exCOPD measuring 11 parameters and found that leukocyte and neutrophil cell count, red cell distribution width, C-reactive protein, neutrophil-to-lymphocyte ratio, and platelet-to-lymphocyte ratio were higher in exCOPD than healthy controls (55). Barta et al. recently found in the sputum that IL-6 and growth-regulated oncogene-α were higher in stCOPD than in non-smoker controls, and the IL-1Rα, RANTES, MIG, BMP-4, BMP-6, GDNF, and Acrp30 were higher in exCOPD than stCOPD (56). Our panel for the Luminex MAGPIX® was a commercially available immuno-oncology panel that showed the mixed phenotype of COPD and lung cancer patients. The plasma of exCOPD patients showed an inflammatory phenotype with the lowest concentration of inhibitory PD-L2, a lower amount of TIM-3 vs. stCOPD, and an increased level of stimulatory CD86 and GITRL vs. smoker controls. The distribution of the defined clusters represents the heterogeneity of the human subjects belonging to either SmHC, stCOPD, exCOPD, or NSCLC categories. These disease-associated heterogenic phenotypes may harbor therapeutic resistance and contribute to the challenging scenarios for the treatment options.

**Figure 9:**
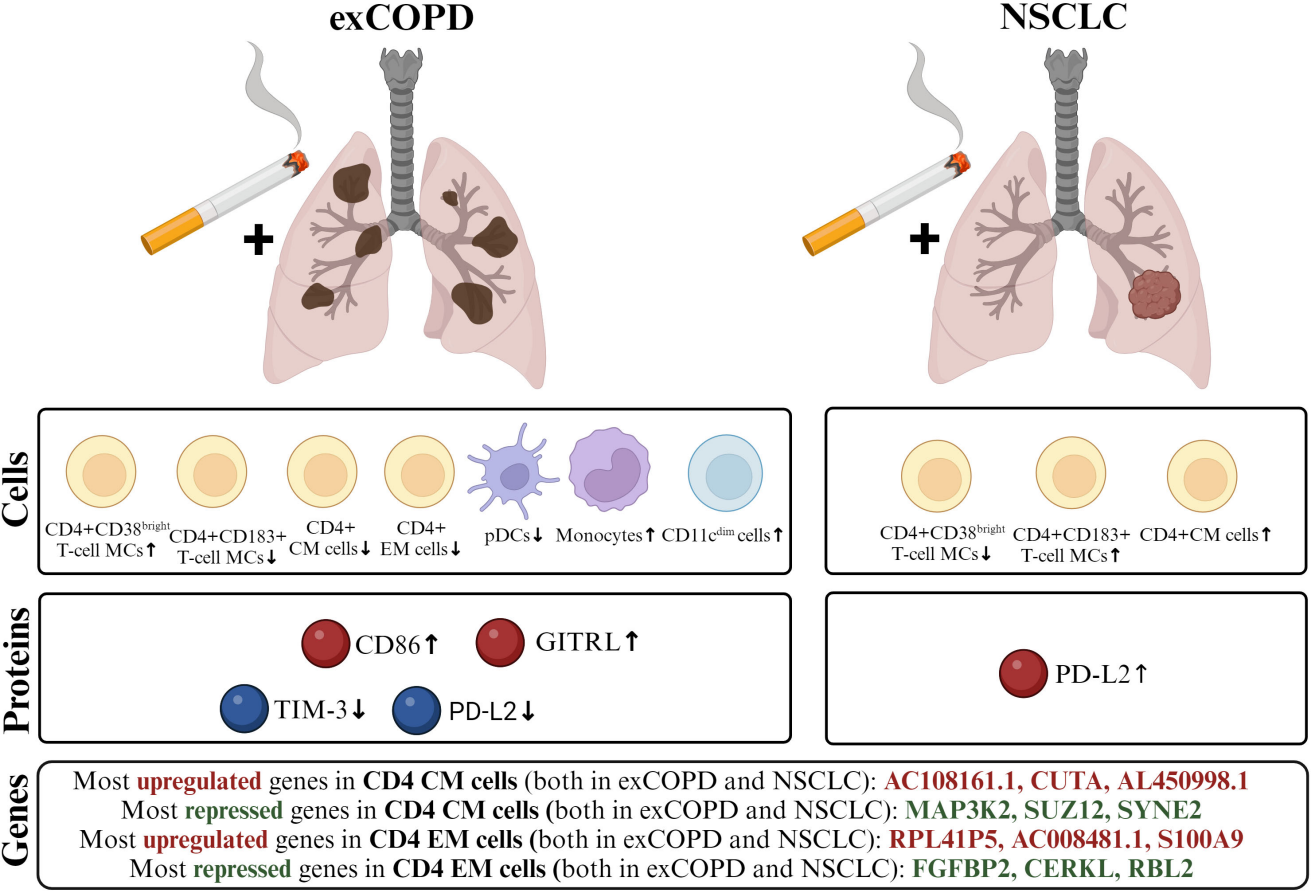
The schematic cartoon of our result focusing on three levels: genes, proteins, and cells. The transcriptome of CD4+ CM and CD4+ EM cells showed a correlation in exCOPD and NSCLC. The three most differentially expressed genes are highlighted. Next, soluble mediators were measured, where CD86 and GITRL were higher in the plasma of exCOPD patients, while TIM-3 and PD-L2 were lower in exCOPD patients. Lastly, single-cell immunophenotyping revealed alterations in the composition of peripheral compartments, where the frequency of the immune subsets was quantified and demonstrated characteristics for exCOPD and NSCLC.

Among others, the authors reviewed how chronic inflammation provides the soil for the development of cancer (57). The authors reviewed previously also how COPD may precondition for the development of lung cancer (5). One of the main messages of the current study is the smoking-related systemic inflammation linking exCOPD and NSCLC demonstrated here as a complex single-cell mass cytometric profile. The authors reported here a complex view of the peripheral immunophenotype with a special focus on the T-cell compartment (**Figure 9**). Freeman et al. published earlier the decrease of CD4+ and CD8+ T cells in the peripheral blood of acute exacerbation COPD patients in agreement with our results (58).

Focusing on T-cell biology, activation markers such as CD38 on CD4 T cells, CD25 on CD4+CD8+ T cells, and CD28 on DN T cells were shown upregulated in exCOPD in conjunction with the drop of the Th1 marker CD183 and chemokine receptor CXCR3 on CD4, CD8, DN, and γ/δ T cells in exCOPD. Similarly, the decrease of CD183 was also shown on cytotoxic CD8+ T cells and DN T cells in NSCLC. The decrease of the co-stimulatory CD27 was also shown on CD4 T cells, CD8 T cells, and CD4+CD8+ T cells in NSCLC. Although the frequency of CD4+ CM and CD4+ EM cells was not the same in exCOPD and NSCLC, their transcriptomic profile showed 0.8 and 0.74 Pearson correlation, respectively. Since CD4+ helper T cells orchestrate the polarization of the adaptive immunity, the high coverage of mRNAs in these memory cells may play a role in the antigen-dependent inflammatory pathways in exCOPD and NSCLC. The authors hypothesize that tobacco smoking-generated inflammation polarizes CD4+ CM and CD4+ EM cells that may contribute to the exacerbation of COPD and impairment of tumor-protective immunity in NSCLC. Taken together, it is hard to estimate which difference in the immunophenotype has the most meaningful clinical importance. Our data may serve as a resource for future studies that will lead to a deeper understanding of the disbalance in immune homeostasis in smoker COPD or NSCLC patients.

Our study has limitations which are as follows: 1) that PBMCs lack a granulocyte compartment which has a significant effect on the chronic inflammation in the studied lung pathologies. 2) Our CyTOF panel consisted of cell surface markers instead of the implementation of subset-specific cytokine/chemokine measurement. 3) We could not investigate tissue-resident immune cells of the affected lung in COPD or NSCLC cases. 4) We could enroll SmHCs with 10 years younger age than the patients. The access to the healthy but aging population is limited with a special focus on the inclusion criteria of the smoking habit. Therefore, we could enroll smoker healthy subjects for the withdrawal of blood from this group. 5) Bacterial or viral infections were not excluded and may influence the immunophenotype of exCOPD or NSCLC. However, according to our knowledge, this is the first study on the deep immunophenotyping of the single-cell heterogeneity in smoker controls, stCOPD, exCOPD, and NSCLC patients with an extension of the transcriptome of CD4+ CM and CD4+ EM cells in the four studied groups. Future studies are warranted to measure the soluble mediators of the identified disease-associated subsets to link serum concentrations with the producing immune subsets.

## Data availability statement

The original contributions presented in the study are publicly available. This data can be found here: https://www.ncbi.nlm.nih.gov/geo/; GSE250254.

## Ethics statement

The study was conducted in accordance with the Declaration of Helsinki, and the protocol (‘Immunophenotyping in COPD and lung cancer’) was approved by the Ethics Committee of the National Public Health Center under the 33815-7/2018/EÜIG Project identification code. The studies were conducted in accordance with the local legislation and institutional requirements. The participants provided their written informed consent to participate in this study.

## Author contributions

NG: Formal analysis, Investigation, Methodology, Visualization, Writing – original draft. JB: Data curation, Methodology, Writing – original draft. PN: Data curation, Investigation, Methodology, Writing – original draft. ES: Investigation, Methodology, Writing – original draft. IB: Methodology, Writing – original draft. JF: Methodology, Writing – original draft. BA: Data curation, Methodology, Visualization, Writing – original draft. ÁZ: Data curation, Formal analysis, Writing – original draft. ZH: Data curation, Formal analysis, Methodology, Writing – original draft. ZC: Data curation, Formal analysis, Writing – original draft. MM: Formal analysis, Methodology, Writing – original draft. GB: Methodology, Writing – original draft. JT: Data curation, Formal analysis, Writing – original draft. LP: Funding acquisition, Resources, Supervision, Writing – original draft. GS: Conceptualization, Data curation, Funding acquisition, Resources, Supervision, Visualization, Writing – original draft.
