# Supplementary figures and images for: Single-cell immunophenotyping revealed the association of CD4+ central and CD4+ effector memory T cells linking exacerbating chronic obstructive pulmonary disease and NSCLC
Source: Front Immunol. 2023 Dec 20;14:1297577. doi: 10.3389/fimmu.2023.1297577 (PMC10770259; doi:10.3389/fimmu.2023.1297577)

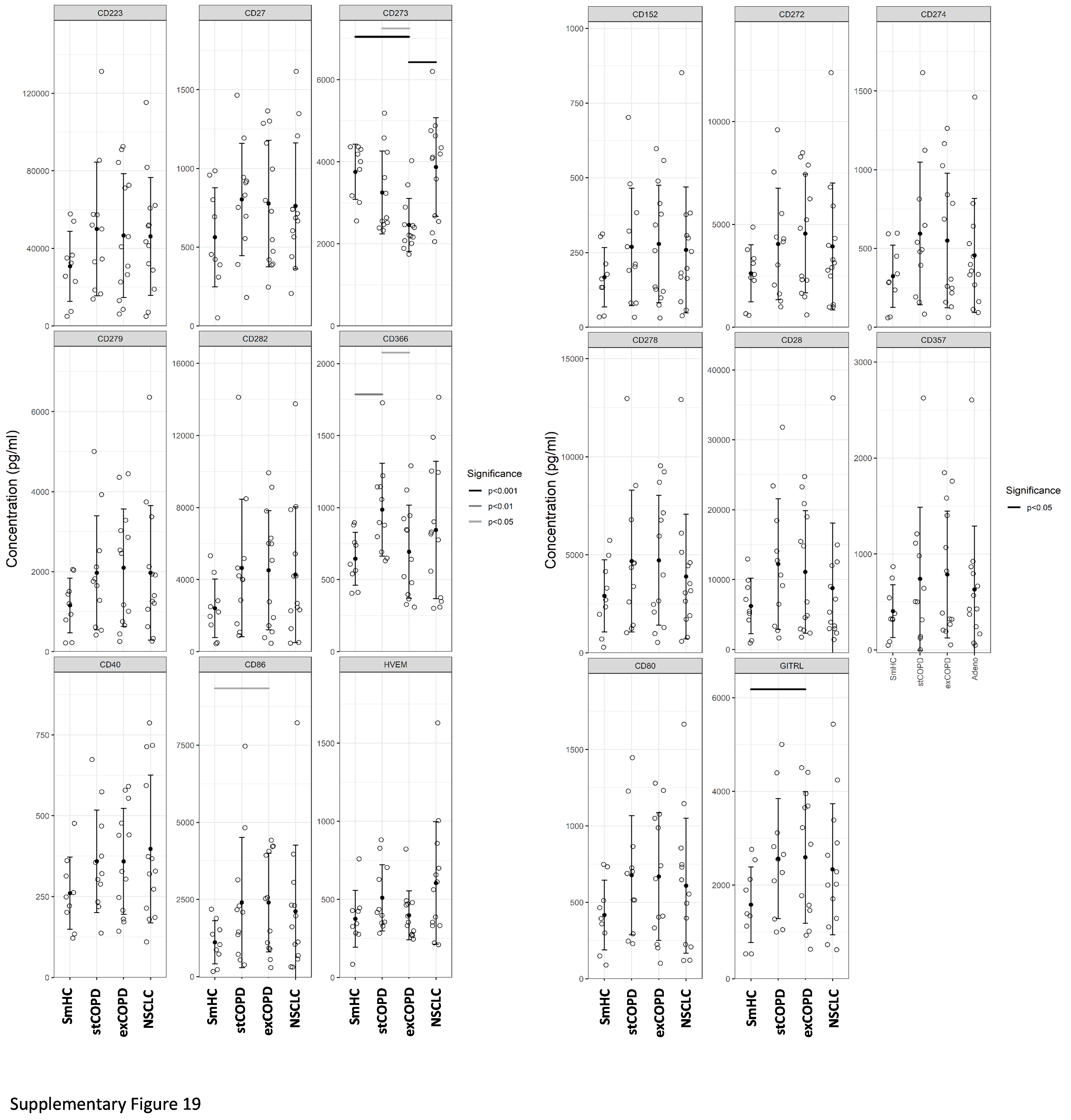

Supplement: Supplementary file 1 [file Image_1.tif]
