# Supplementary material for: Single-cell immunophenotyping revealed the association of CD4+ central and CD4+ effector memory T cells linking exacerbating chronic obstructive pulmonary disease and NSCLC
Source: Front Immunol. 2023 Dec 20;14:1297577. doi: 10.3389/fimmu.2023.1297577 (PMC10770259; doi:10.3389/fimmu.2023.1297577)
Supplement: Supplementary file 2 [file Presentation_1.pdf]

*Supplementary Material*

# Single-cell immunophenotyping revealed the association of CD4+ central and CD4+ effector memory T cells linking exacerbating chronic obstructive pulmonary disease and NSCLC

**Nikolett Gémes[1,2], József Á. Balog [1], Patrícia Neuperger[1,2], Erzsébet Schlegl[3], Imre Barta[3], János Fillinger[3], Balázs Antus[3], Ágnes Zvara[1], Zoltán Hegedűs[4,5], Zsolt Czimmerer[6], Máté Manczinger [7,8], Gergő Mihály Balogh[7,8], József Tóvári[9], László G. Puskás [1,10,11,*], and Gábor J. Szebeni[1,12,13,*]**

*** Correspondence:**
Laszlo G. Puskas (laszlo@avidinbiotech.com), Gabor J. Szebeni (szebeni.gabor@brc.hu)
*These authors contributed equally to this work and share senior authorship

## 1. Supplementary Tables

### 1.1 Supplementary Table 1

| | SmHC | stCOPD | exCOPD | NSCLC |
|---|---|---|---|---|
| **Subjects (n)** | 9 | 11 | 13 | 14 |
| **Sex (male/female, n)** | 3/6 | 4 / 7 | 5 / 8 | 6 / 8 |
| **Age (years, mean ± SEM)** | 55 ± 0.35 | 66.7 ± 1.7 | 68.3 ± 2.8 | 68.7 ± 2.3 |
| **Smoking (pack-years)** | min. 5 | 38.1 ± 6.5 | 43.1 ± 9.4 | 14.1 ± 3.1 |
| **GOLD stages (n, %)$** | | | | |
| **I-II.** | - | 3 (27) | 2 (15) | - |
| **III-IV.** | - | 7 (64) | 9 (69) | - |

| | | | | |
|---|---|---|---|---|
| **Tumor stages (n, %)#** | | | | |
| **I.** | - | - | - | 2 (14) |
| **II.** | - | - | - | 0 (0) |
| **III.** | - | - | - | 4 (29) |
| **IV.** | - | - | - | 6 (43) |
| **Pulmonary function** | | | | |
| **FVC (L)** | - | 1.85 ± 0.3 | 1.75 ± 0.09 | 2.3 ± 0.21 |
| **$FEV_1$ (L)** | - | 0.96 ± 0.16 | 0.81 ± 0.09 | 1.54 ± 0.16 |
| **$FEV_1$/FVC (%)** | - | 52.2 ± 5.1 | 44.9 ± 3.6 | 66.3 ± 2.4 |
| **Blood gases** | | | | |
| **$PaCO_2$ (kPa)** | - | 5.3 ± 1.04 | 6.93 ± 1.12 | - |
| **$PaO_2$ (kPa)** | - | 7.09 ± 0.5 | 7.99 ± 0.40 | - |
| **Laboratory data** | | | | |
| **WBC ($\times 10^9$/L)** | - | 11.4 ± 0.9 | 11.7 ± 0.84 | 11.5 ± 1.8 |
| **CRP (mg/L)** | - | 60.6 ± 24.1 | 53.7 ± 17.1 | 50.9 ± 20.0 |

**Supplementary Table 1.** The demographic characteristic and clinical parameters of the enrolled patients into the study. Before the withdrawal of the peripheral blood written informed consent was signed by the patients. Blood, the purified PBMCs and plasma were processed anonymously. Notes: Data are presented as mean ± SEM. $In one stable and two exCOPD patients no information was obtained. #In two patients adequate staging was not available. SmHc were interviewed for a minimum5 pack-year smoking history. Abbreviations: SmHc: smoker healthy controls; COPD: chronic obstructive pulmonary disease; stCOPD: stable COPD; exCOPD: acute exacerbation of COPD; NSCLC: non-small cell lung cancer; FVC, forced vital capacity; $FEV_1$, forced expiratory volume in one second; $PaCO_2$, arterial carbon dioxide tension; $PaO_2$, arterial oxygen tension; GOLD, global initiative for chronic obstructive pulmonary disease; CRP, C-reactive protein; WBC, white blood cells

## 1.2 Supplementary Table 2

| Antibody (Fluidigm) | Clone | Metal tag |
|---|---|---|
| Anti-Human CD45 | HI-30 | $^{89}Y$ |
| Anti-Human CD196/CCR6 | G034E3 | $^{141}Pr$ |
| Anti-Human CD19 | HIB19 | $^{142}Nd$ |
| Anti-Human CD127/IL-7Ra | A019D5 | $^{143}Nd$ |
| Anti-Human CD38 | HIT2 | $^{144}Nd$ |
| Anti-Human IgD | IA6-2 | $^{146}Nd$ |
| Anti-Human CD11c | Bu15 | $^{147}Sm$ |
| Anti-Human CD16 | 3G8 | $^{148}Nd$ |
| Anti-Human CD194/CCR4 | L29IH4 | $^{149}Sm$ |
| Anti-Human CD123/IL-3R | 6H6 | $^{151}Eu$ |
| Anti-Human TCRgd | 11F2 | $^{152}Sm$ |
| Anti-Human CD185/CXCR5 | RF8B2 | $^{153}Eu$ |
| Anti-Human CD3 | UCHT1 | $^{154}Sm$ |
| Anti-Human CD45RA | HI100 | $^{155}Gd$ |
| Anti-Human CD27 | L128 | $^{158}Gd$ |
| Anti-Human CD28 | CD28.2 | $^{160}Gd$ |
| Anti-Human CD66b | 80H3 | $^{162}Dy$ |
| Anti-Human CD183/CXCR3 | G02H7 | $^{163}Dy$ |
| Anti-Human CD161 | HP-3G10 | $^{164}Dy$ |
| Anti-Human CD45RO | UCHL1 | $^{165}Ho$ |
| Anti-Human CD24 | ML5 | $^{166}Er$ |
| Anti-Human CD197/CCR7 | G043H7 | $^{167}Er$ |
| Anti-Human CD8 | SK1 | $^{168}Er$ |
| Anti-Human CD25 | 2A3 | $^{169}Tm$ |
| Anti-Human CD20 | 2H7 | $^{171}Yb$ |
| Anti-Human HLA-DR | L243 | $^{173}Yb$ |
| Anti-Human CD4 | SK3 | $^{174}Yb$ |
| Anti-Human CD14 | M5E2 | $^{175}Lu$ |
| Anti-Human CD56 | NCAM16.2 | $^{176}Yb$ |

**Supplementary Table 2.** The list of the antibodies, name of the target, the clone and the metal tag used in the Human Immune Monitoring panel.

## 1.3 Supplementary Table 3

**Supplementary Table 3.** *See the attached MS Excel sheet.* Data of the CD4+ EM and CD4+ CM samples for RNAseq, and the results of RNAseq; first layer: sample description; second layer: list of

differentially expressed genes in CD4+ CM, third layer: list of differentially expressed genes in CD4+ EM. Green coloration refers to downregulation, Red coloration refers to induction of gene expression

## 1.4 Supplementary Table 4

**Supplementary Table 4.** *See the attached pdf file.* The list of the proteins measured in the plasma of the human subjects enrolled in the study. The Luminex MagPix multiplex assay was used as described in the Materials and Methods section. Short name, full name, alternative name of the analyzed proteins, gene name, UniProt ID and the range of detection (pg/ml) are listed.

# 2. Supplementary Figures

## 2.1 Supplementary Figure 1

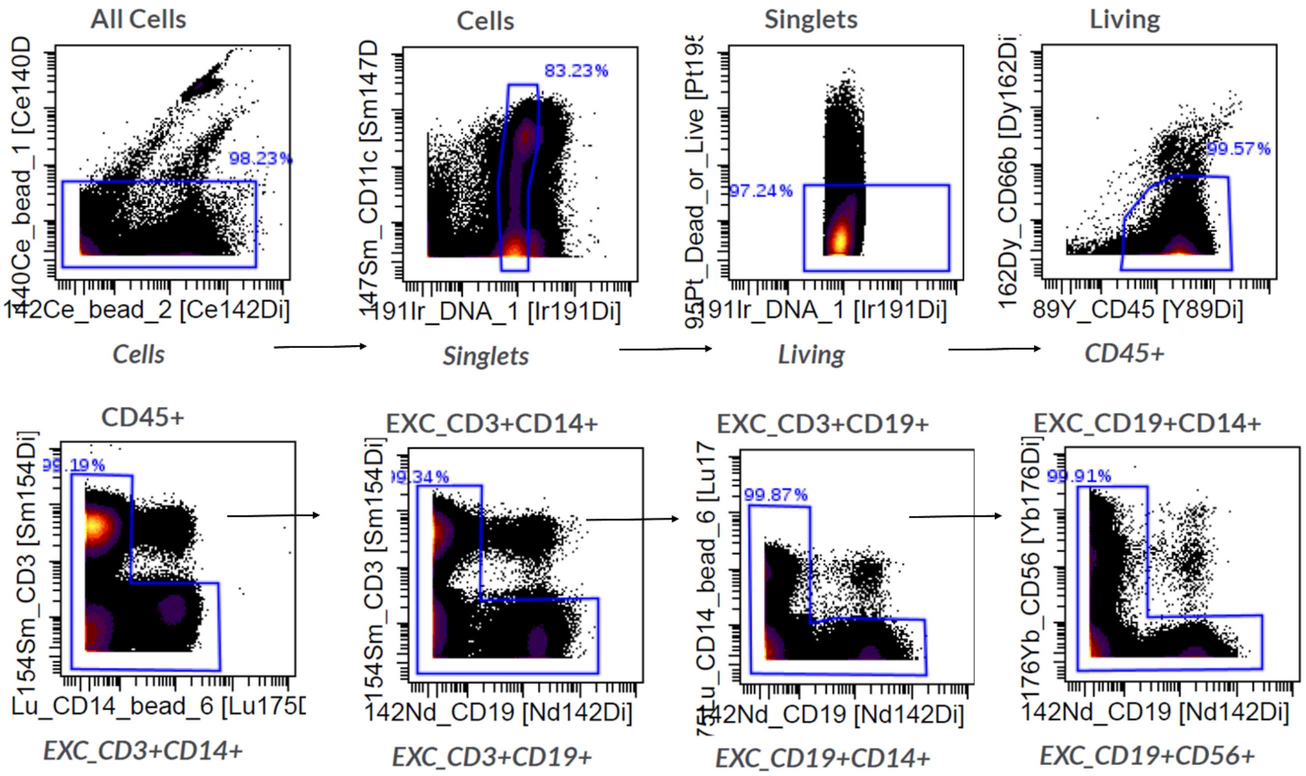


**Supplementary Figure 1.** Manual gating strategy was used to define cells, singlets, living cells, CD45+ cells. Cell populations were excluded which were CD+3CD14+, CD3+CD19+, CD19+CD14+, and CD19+CD56+. The gating hierarchy is from left to right as the title of the dot plots show the parental population. The subsequent analysis was performed following compensation as described in the Materials and Methods.

## 2.2 Supplementary Figure 2

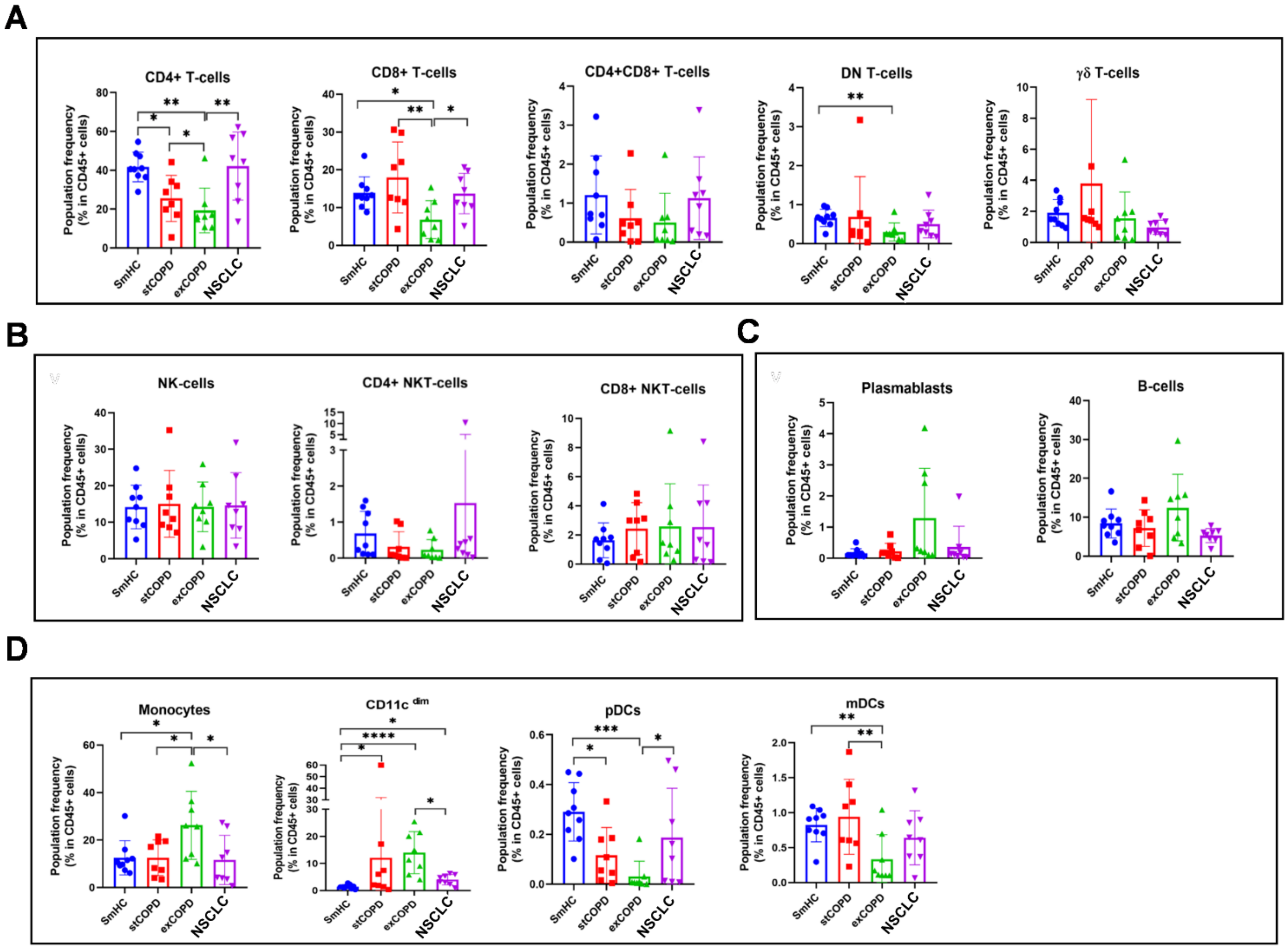


**Supplementary Figure 2.** The population frequency of the fourteen subtypes as a percentage of CD45+ living singlets. (A) T-cell subtypes were analyzed, such as CD4+, CD8+, CD4+CD8+, DN (CD4-CD8-), and γ/δ T-cells. (B) NK cell subtypes: NK, CD4+ NKT, CD8+ NKT cells were analyzed. (C) Plasmablasts and B-cells were analyzed. (D) Myeloid cells, such as monocytes, CD11c$^{dim}$, pDCs, and mDCs were quantified. p *<0.05, **<0.01, ***<0.001

## 2.3 Supplementary Figure 3

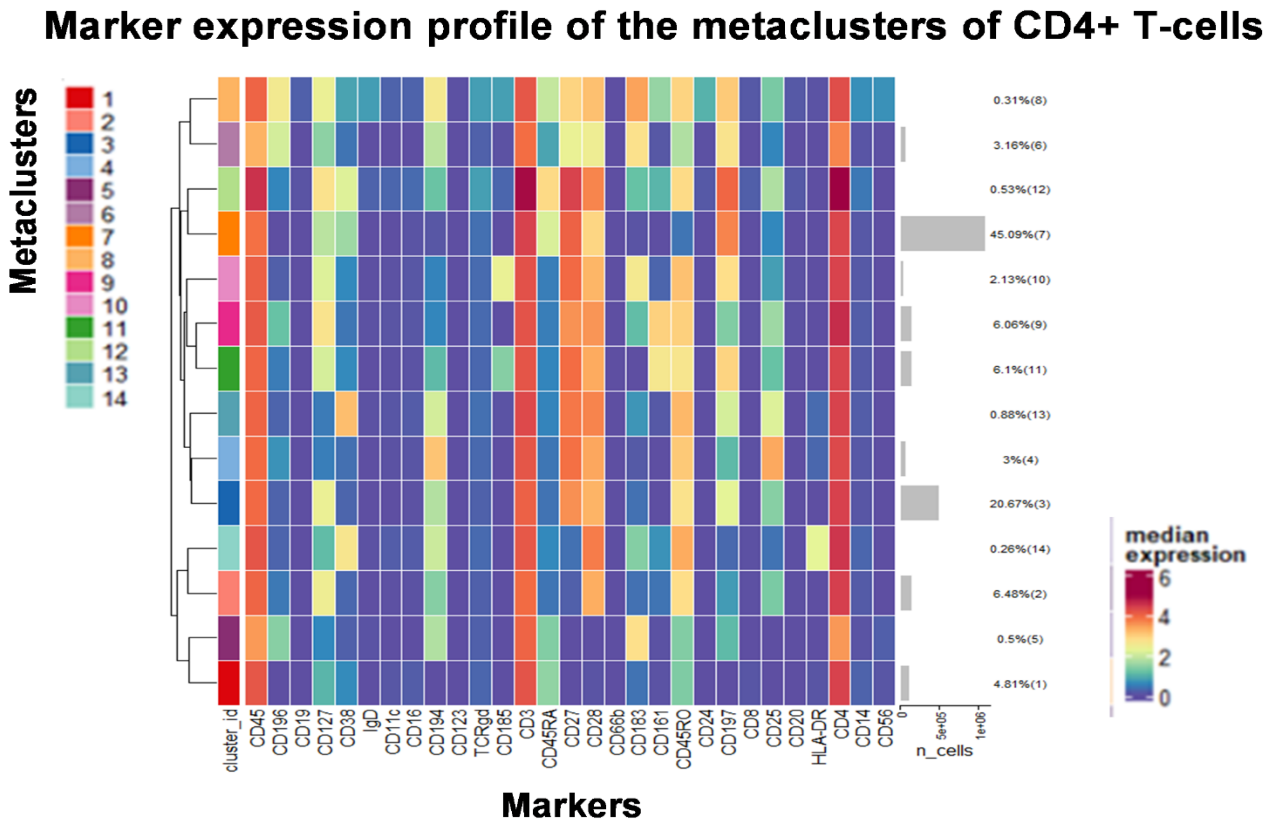


**Supplementary Figure 3.** The marker expression profile of the metaclusters of CD4+ T-cells. The color code on the right side (from blue to red) is proportional with the expression intensity.

### 2.4 Supplementary Figure 4

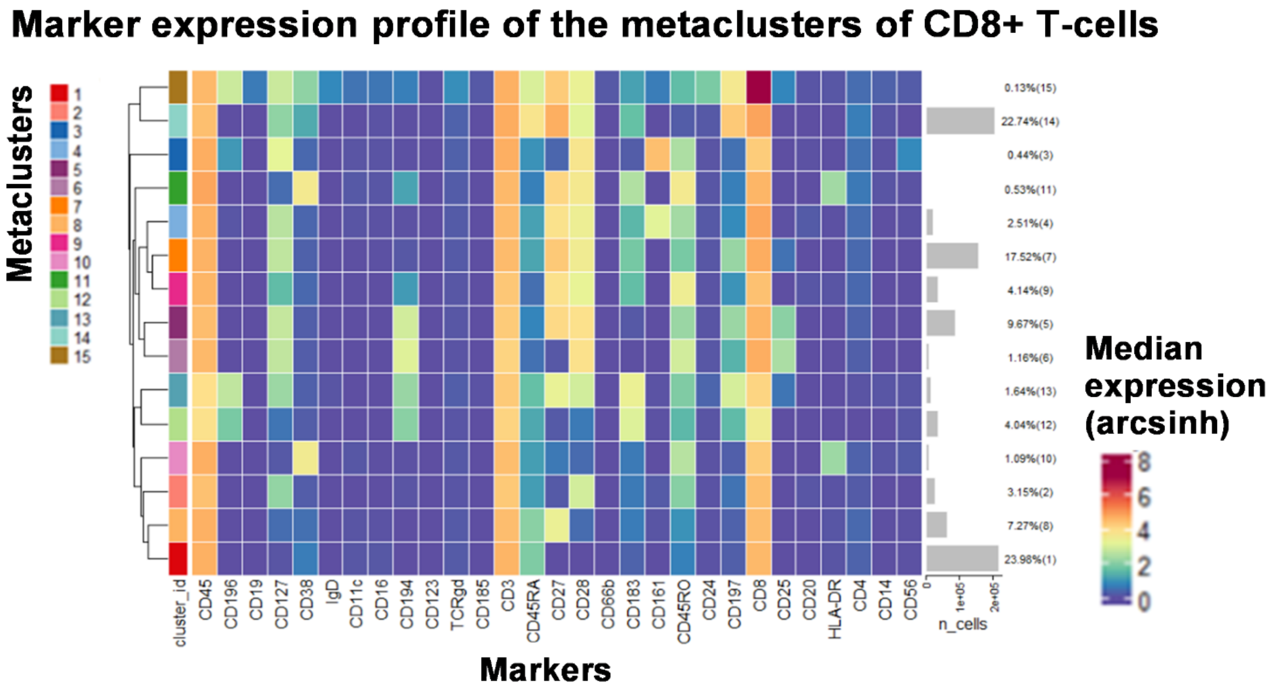


**Supplementary Figure 4.** The marker expression profile of the metaclusters of CD8+ T-cells. The color code on the right side (from blue to red) is proportional with the expression intensity.

### 2.5 Supplementary Figure 5

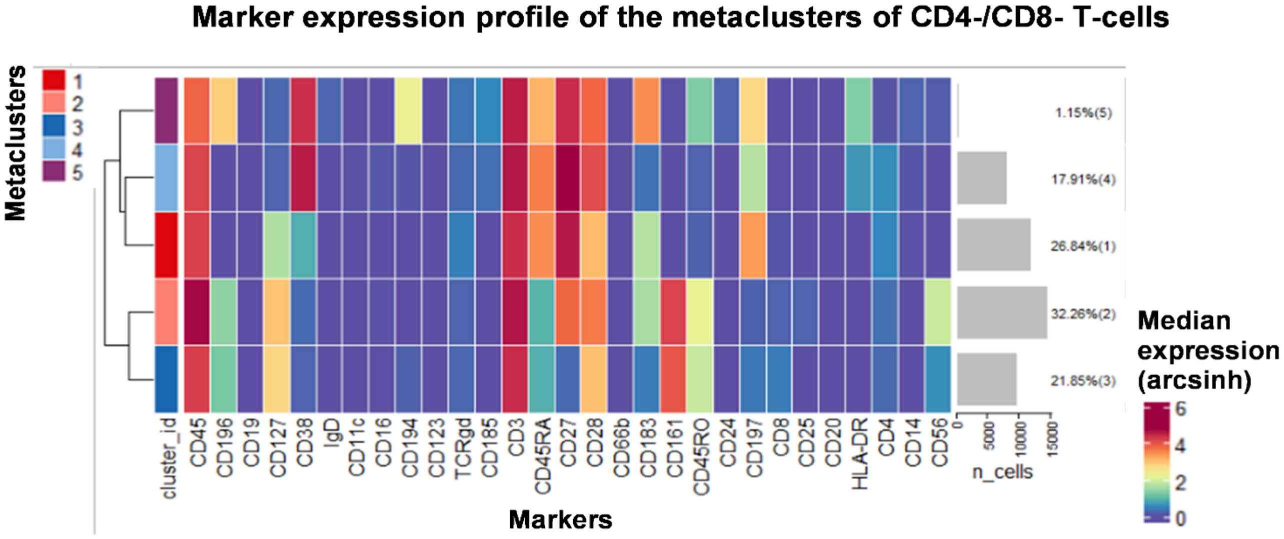


**Supplementary Figure 5.** The marker expression profile of the metaclusters of CD4-CD8- T-cells. The color code on the right side (from blue to red) is proportional with the expression intensity.

## 2.6 Supplementary Figure 6

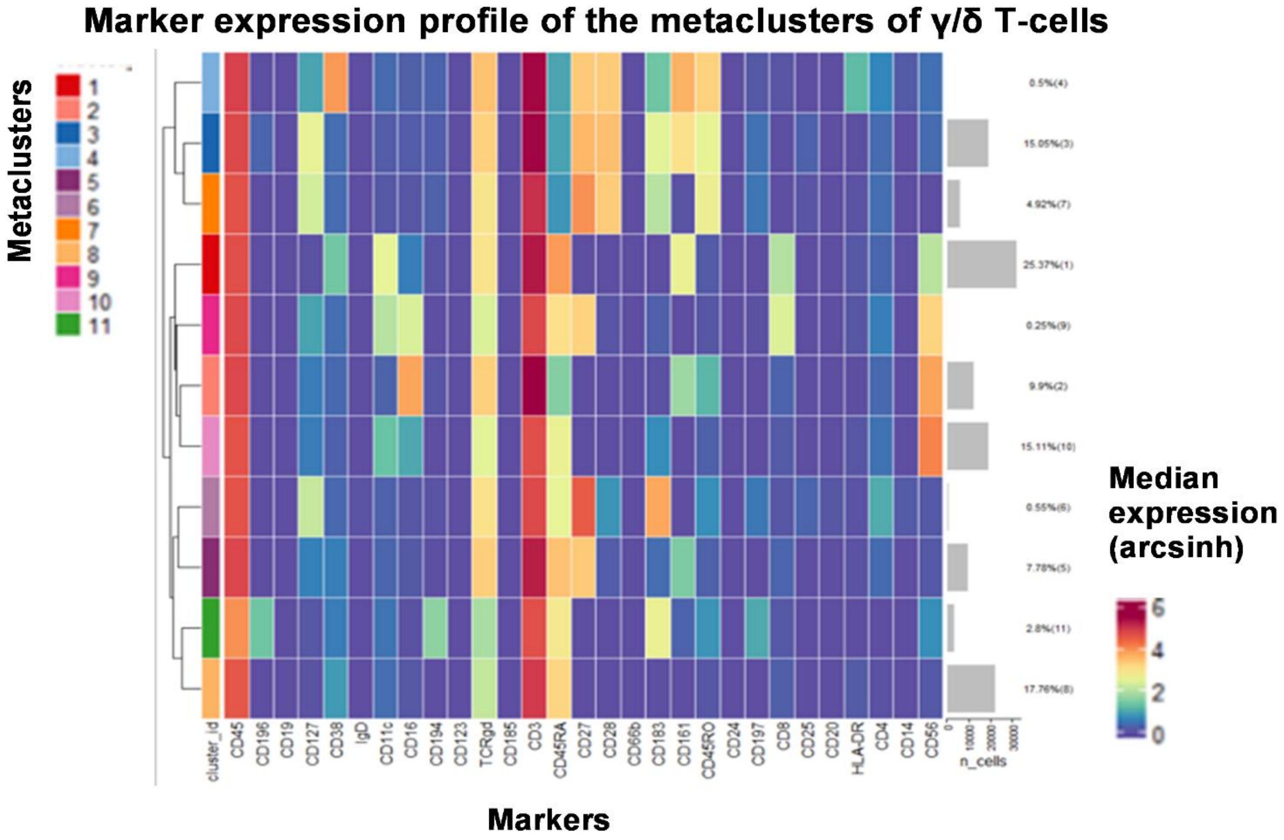


**Supplementary Figure 6.** The marker expression profile of the metaclusters of γ/δ T-cells. The color code on the right side (from blue to red) is proportional with the expression intensity.

## 2.7 Supplementary Figure 7

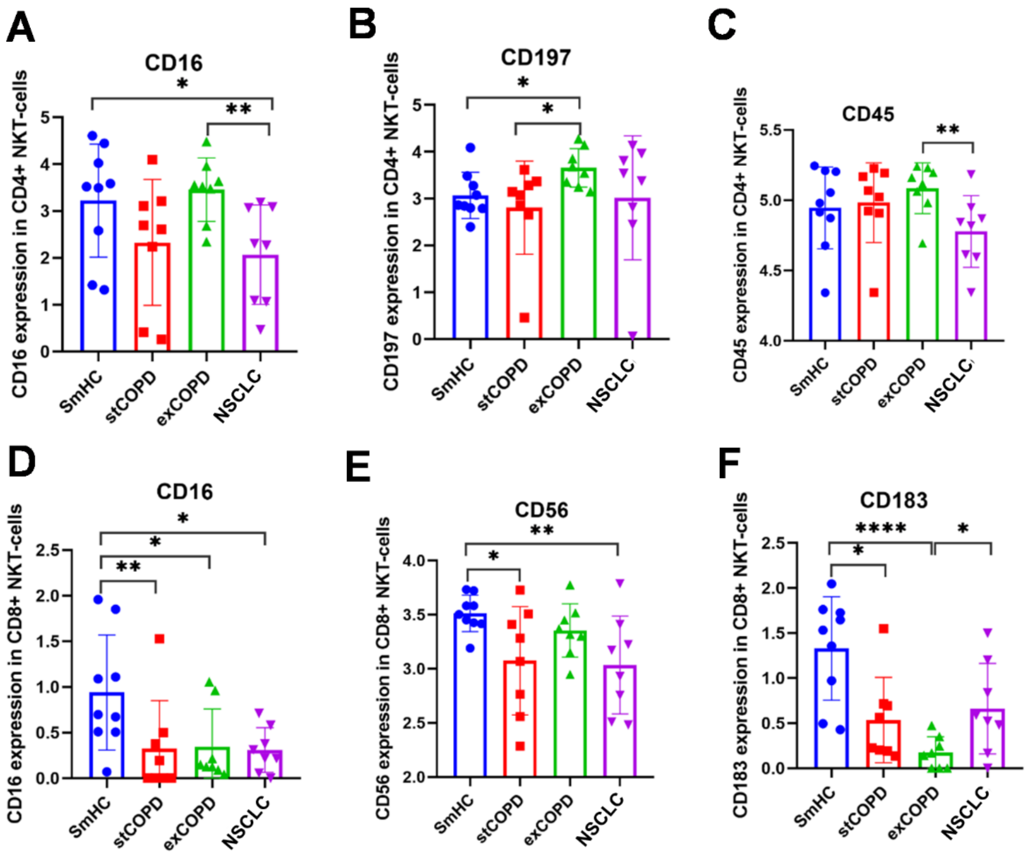


**Supplementary Figure 7.** The marker expression profile of the (A-C) CD4+ NKT cells or (D-F) CD8+ NKT cells. p *<0.05, **<0.01, ***<0.001

## 2.8 Supplementary Figure 8

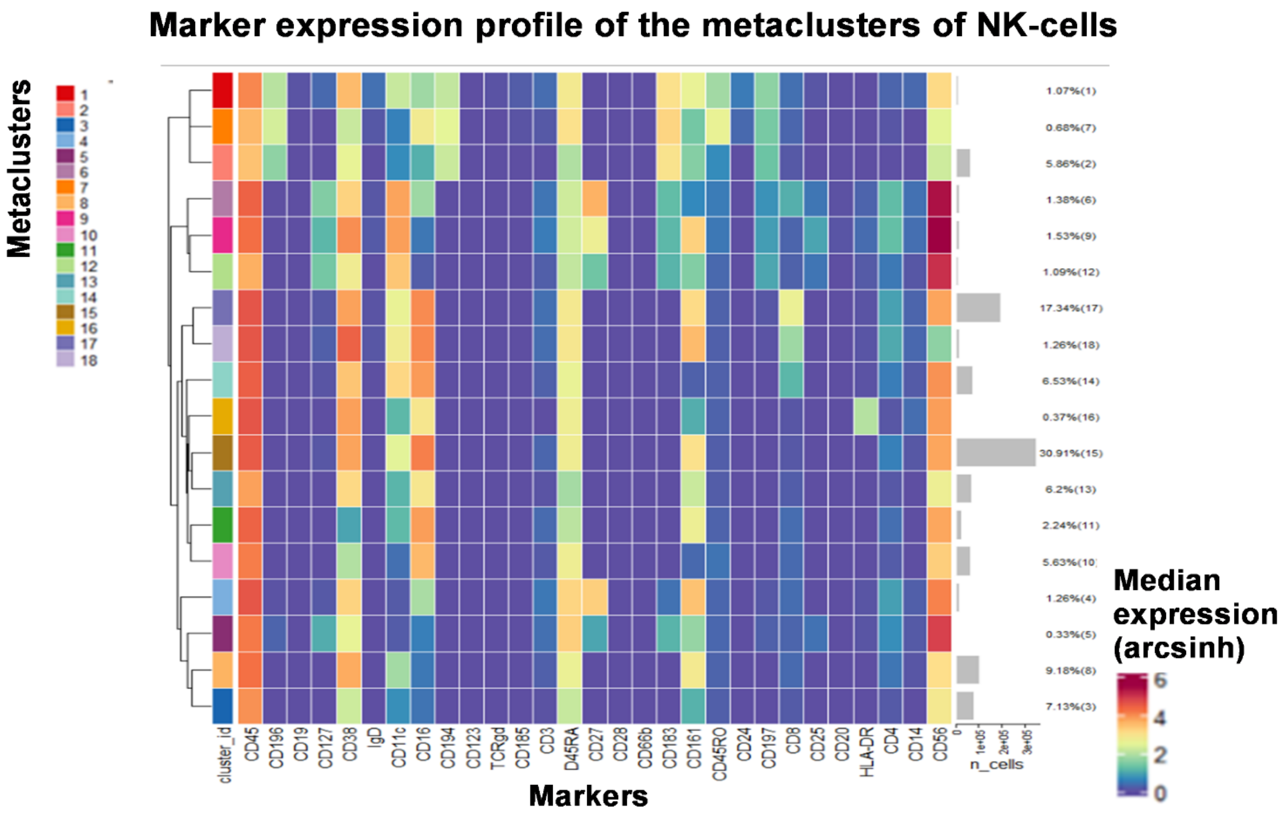


**Supplementary Figure 8.** The marker expression profile of the metaclusters of NK-cells. The color code on the right side (from blue to red) is proportional with the expression intensity.

## 2.9 Supplementary Figure 9

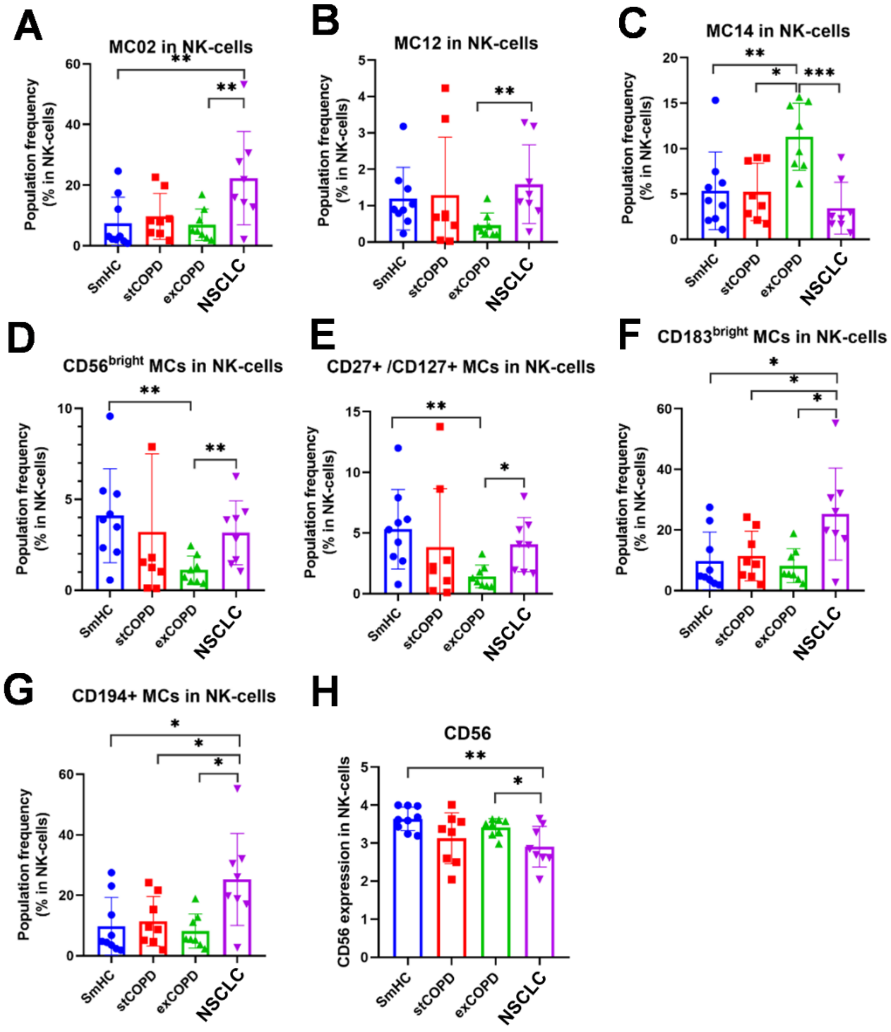


**Supplementary Figure 9.** The population frequency of NK metaclusters (A-G) and CD56 marker expression profile of the NK-cells. p *<0.05, **<0.01, ***<0.001

**2.10 Supplementary Figure 10**

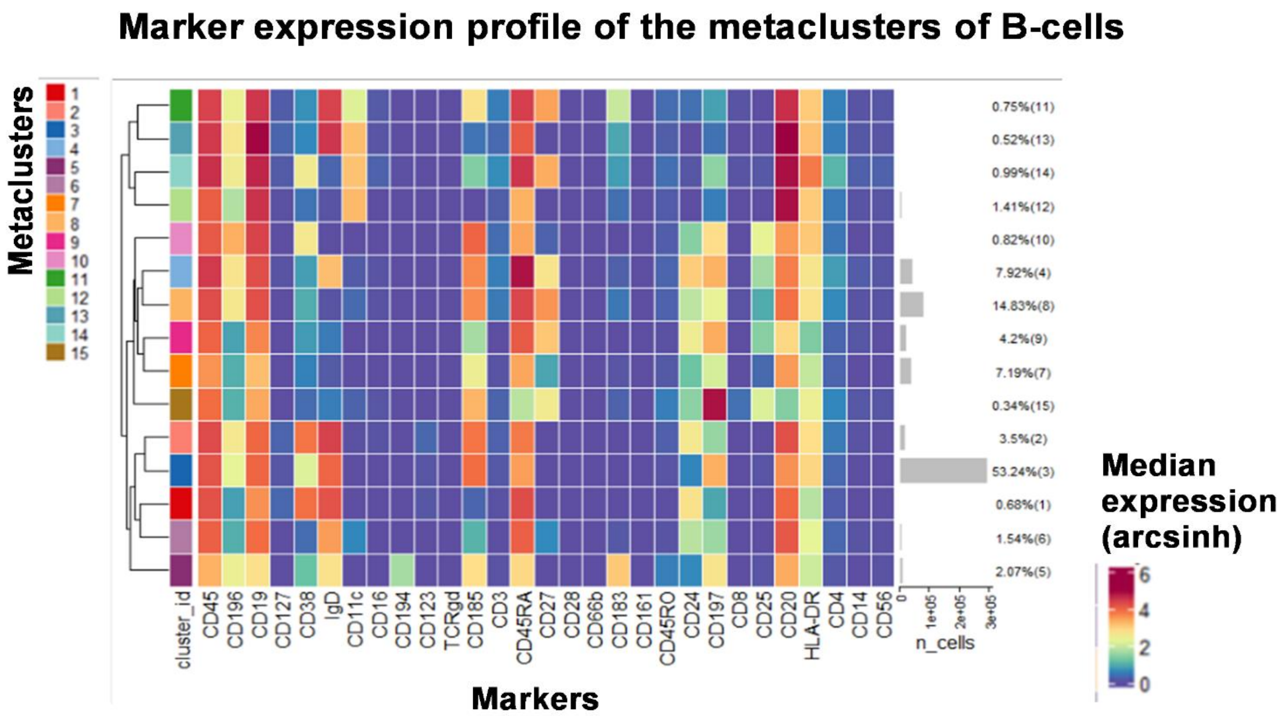


**Supplementary Figure 10.** The marker expression profile of the metaclusters of B-cells. The color code on the right side (from blue to red) is proportional with the expression intensity.

## 2.11 Supplementary Figure 11

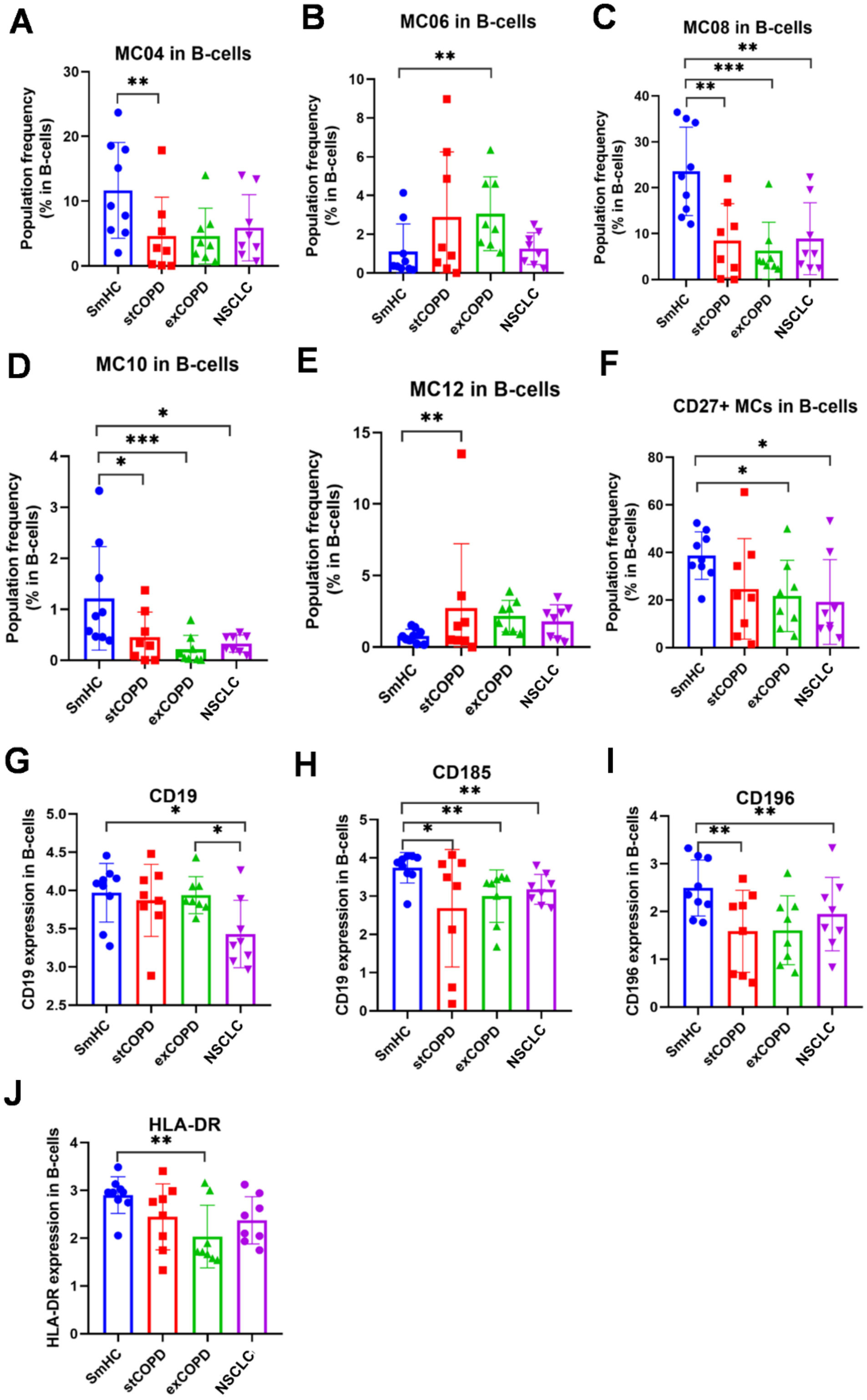


**Supplementary Figure 11.** The population frequency of B-cell metaclusters (A-F) and marker expression profile (G-J) of the B-cells. p *<0.05, **<0.01, ***<0.001

## 2.12 Supplementary Figure 12

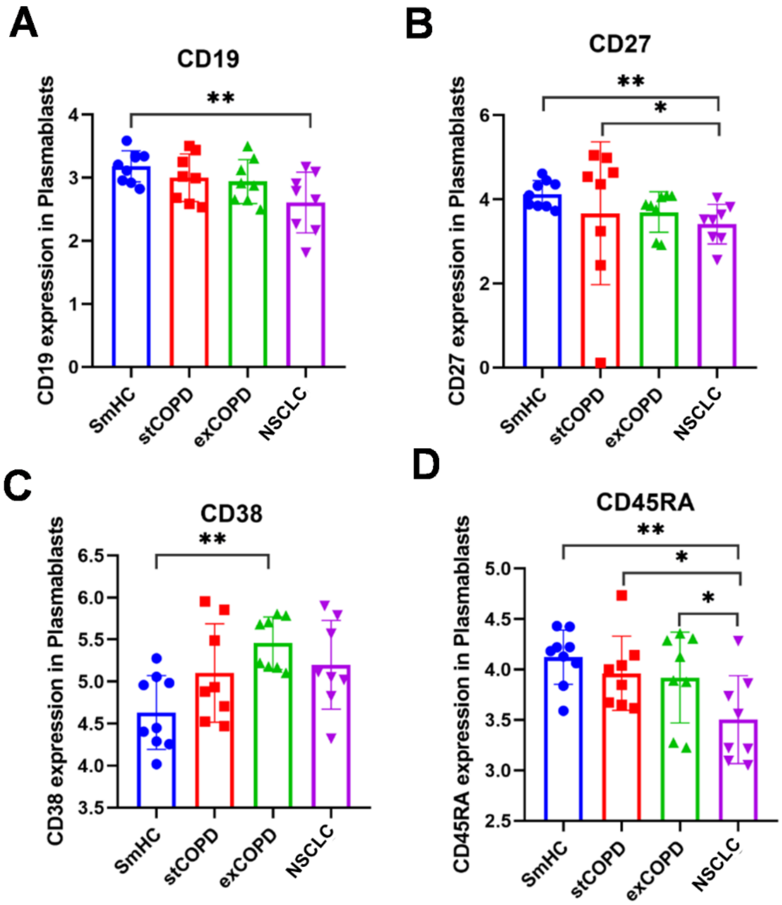


**Supplementary Figure 12. The** marker expression profile of the plasmablasts. p *<0.05, **<0.01, ***<0.001

## 2.13 Supplementary Figure 13

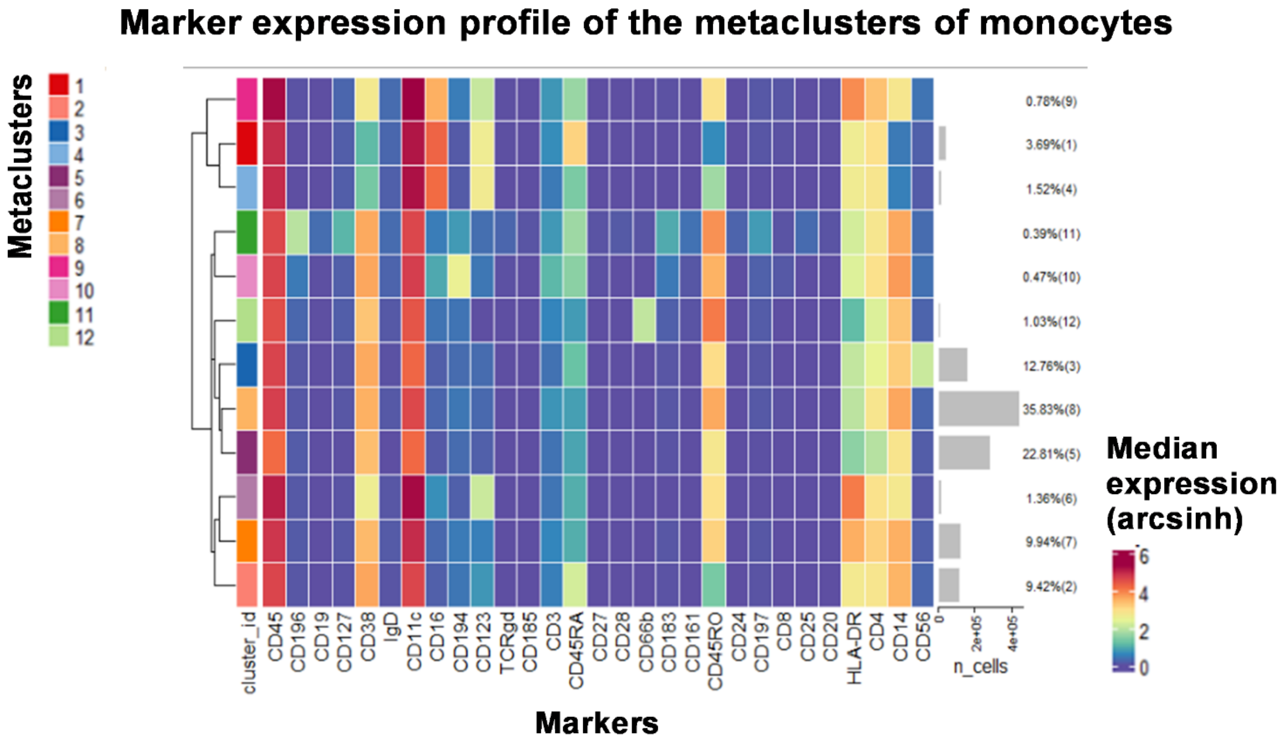


**Supplementary Figure 13.** The marker expression profile of the metaclusters of monocytes. The color code on the right side (from blue to red) is proportional with the expression intensity.

## 2.14 Supplementary Figure 14

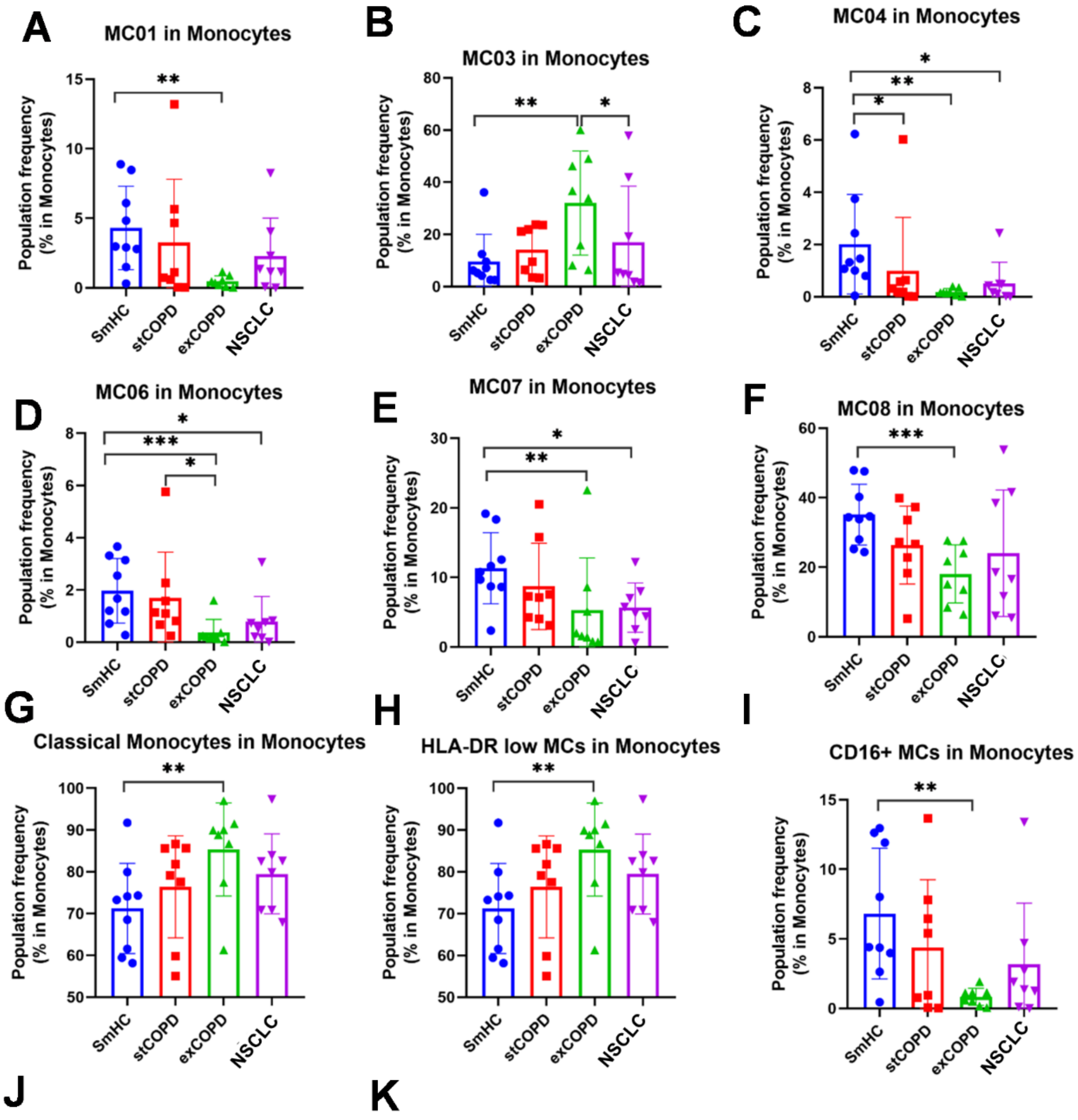


**Supplementary Figure 14.** The population frequency of monocyte metaclusters (A-I), (classical monocytes are merged MC02, MC05, MC08, MC10, MC11, MC12), and marker expression profile (J-K) of the monocytes. p *<0.05, **<0.01, ***<0.001

### 2.15 Supplementary Figure 15

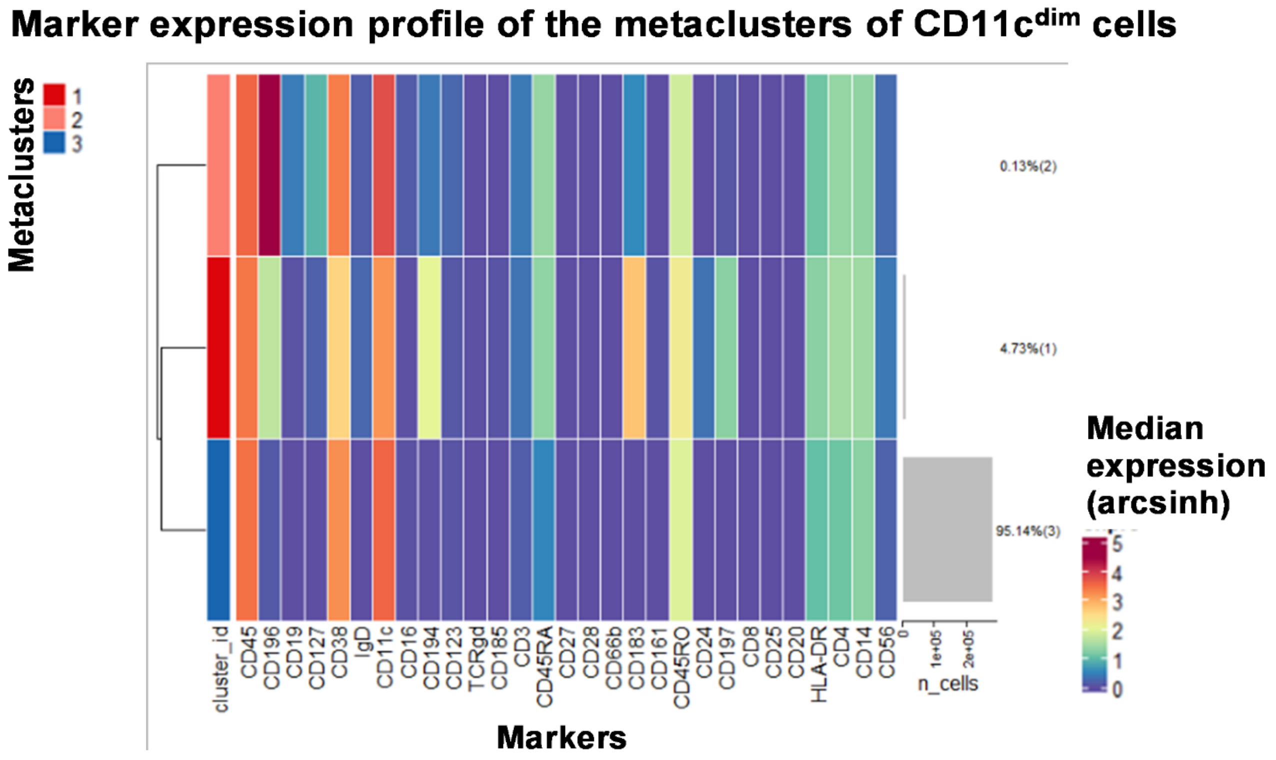


**Supplementary Figure 15.** The marker expression profile of the metaclusters of CD11c$^{dim}$ cells. The color code on the right side (from blue to red) is proportional with the expression intensity.

## 2.16 Supplementary Figure 16

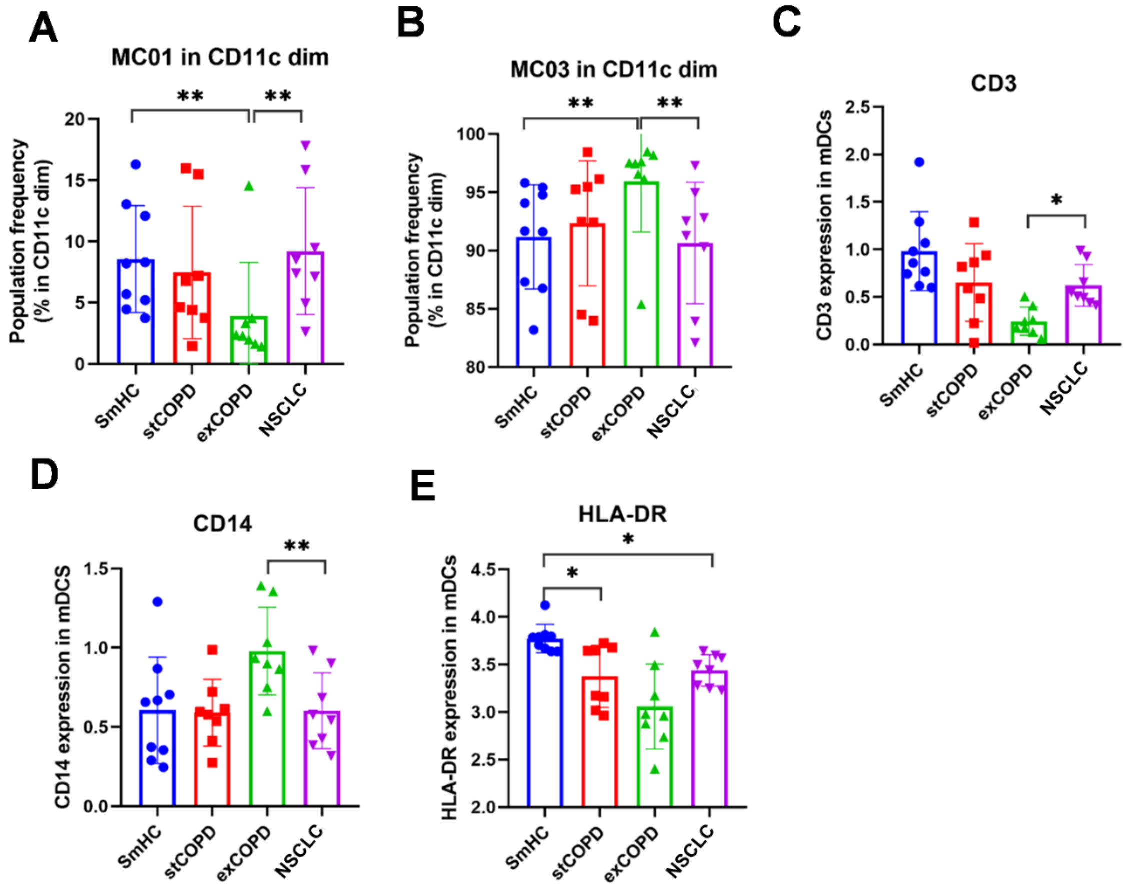


**Supplementary Figure 16.** The population frequency of CD11c$^{dim}$ cells (A-B) and marker expression profile (C-E) of the mDCs. p *<0.05, **<0.01, ***<0.001

## 2.17 Supplementary Figure 17

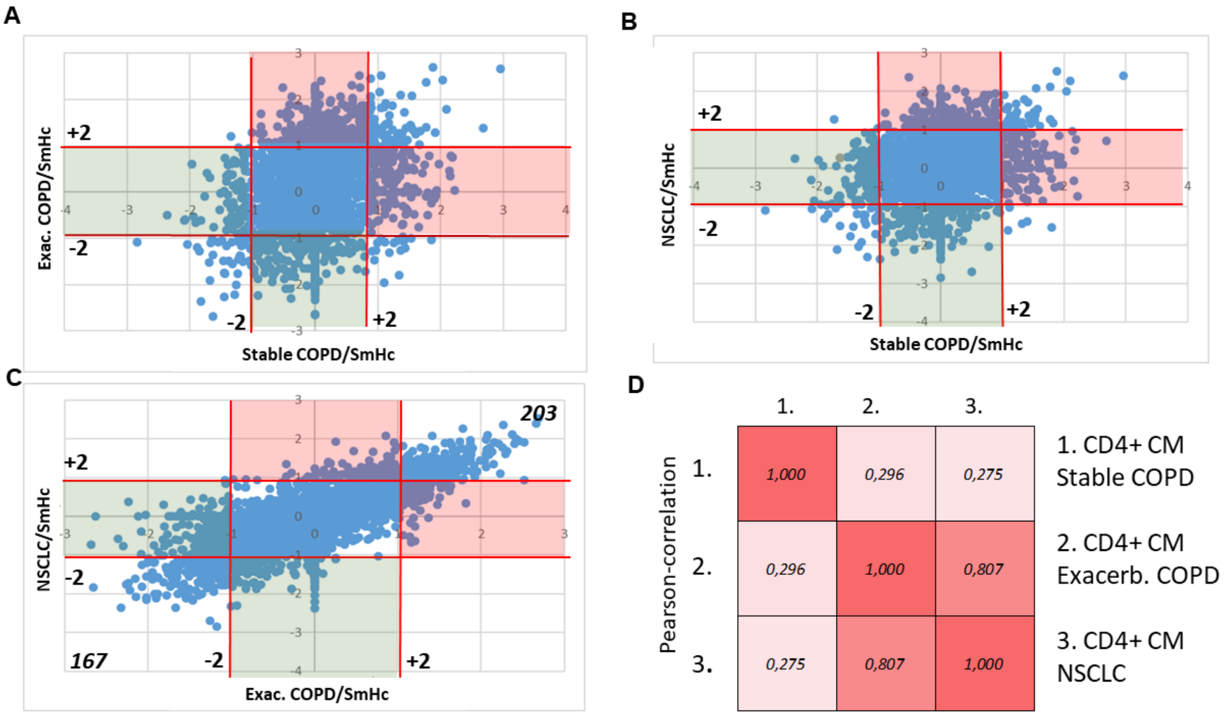


**Supplementary Figure 17.** The schematic cartoons of the transcripts from the RNAseq in CD4+ CM cells in relation to (A) exacerbating COPD and stable COPD, (B) NSCLC and stable COPD, (C) NSCLC and exacerbating COPD. 203 common genes showed increased expression and 167 common genes showed downregulation in NSCLC and exacerbating COPD.  (D) Pearson-correlation showed 0.807 correlation of the transcriptome of NSCLC with exacerbating COPD-derived CD4+ CM cells.

## 2.18 Supplementary Figure 18

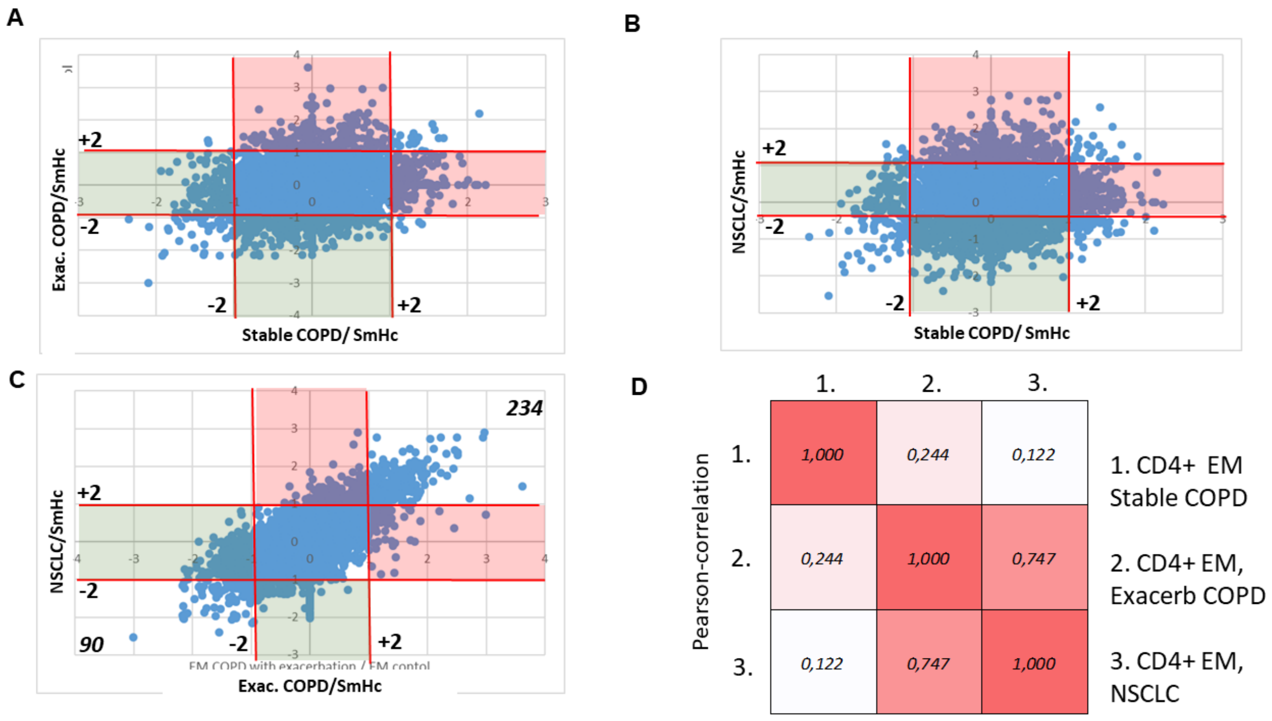


**Supplementary Figure 18.** The schematic cartoons of the transcripts from the RNAseq in CD4+ EM cells in relation to (A) exacerbating COPD and stable COPD, (B) NSCLC and stable COPD, (C) NSCLC and exacerbating COPD. 234 common genes showed increased expression and 90 common genes showed downregulation in NSCLC and exacerbating COPD. (D) Pearson-correlation showed 0.747 correlation of the transcriptome of NSCLC with exacerbating COPD-derived CD4+ EM cells.

## 2.19 Supplementary Figure 19

**The Supplementary Figure 19 is attached in MS Excel file.**

**Supplementary Figure 19.** *See the attached MS Excel sheet.* Scatter plot diagrams shows the concentration values (pg/ml) of the analyzed soluble mediators in the patient-derived plasma samples. SmHC = smoker healthy control, stCOPD = stable COPD, exCOPD = exacerbating COPD, NSCLC = non-small cell lung cancer. The figures were created in R. Significance is shown on the right side of the figures and labeled with gray lines between the corresponding samples. The experimental detail is found in the Materials and Methods section.
