# Supplementary material for: Single-cell immunophenotyping revealed the association of CD4+ central and CD4+ effector memory T cells linking exacerbating chronic obstructive pulmonary disease and NSCLC
Source: Front Immunol. 2023 Dec 20;14:1297577. doi: 10.3389/fimmu.2023.1297577 (PMC10770259; doi:10.3389/fimmu.2023.1297577)
Supplement: Supplementary file 3 [file Presentation_2.pdf]

Supplementary File 1

Codes for the analyses presented on Figure 7 and 8

Gergő Mihály Balogh

2022-04-19

Introduction

The following file contains all the R codes needed for the reproduction of the Gene Set Enrichment Analysis and the clustering of samples based on their cytokine composition (Figure 7 and 8).

Loading libraries and functions

```
library(magrittr)
library(purrr)
library(XML)
library(fastmatch)
library(dplyr)
library(reshape2)
library(ggplot2)
library(cowplot)
library(stringr)
library(ggsci)
library(pheatmap)

PerformGSEA <- function(gsea_path =
"/home/workstation/Programok/GSEA_Linux_4.1.0/gsea-cli.sh", gsea_input,
clsfile, gmx, chip, outdir) {
  cmd <- paste0(gsea_path, " GSEA -res ", gsea_input, " -cls ", clsfile, " -
gmx ", gmx, " -collapse Collapse -mode Max_probe -norm meandiv -nperm 1000 -
permute gene_set -rnd_type no_balance -scoring_scheme weighted -rpt_label
my_analysis -metric Signal2Noise -sort real -order descending -chip ", chip,
" -create_gcts false -create_svgs false -include_only_symbols true -make_sets
true -median false -num 100 -plot_top_x 20 -rnd_seed 149 -save_rnd_lists
false -set_max 500 -set_min 15 -zip_report false -out ", outdir)
  system(cmd, invisible = TRUE)
}

GetMutualHits <- function(x, y, sametrends = TRUE, simplify = TRUE) {
  mutual_genesets <- intersect(x$GENESET, y$GENESET)
  x <- x[fmatch(mutual_genesets, x$GENESET), ]
```

```
  y <- y[fmatch(mutual_genesets, y$GENESET), ]

  if(sametrends) {
    lglvctr <- (x$NES > 0 & y$NES > 0) | (x$NES < 0 & y$NES < 0)
    x <- x[lglvctr, ]
    y <- y[lglvctr, ]
  }
  if(simplify) {
    cols_to_keep <- c("GENESET", "ES", "NES", "FDR")
    left_join(x[, cols_to_keep], y[, cols_to_keep], by = "GENESET")
  } else {
    list(x, y)
  }
}

zscore <- function(x) (x - mean(x))/sd(x)

CreateBreaks2 <- function(minval, maxval, n = 10) {
  abs_maxval <- c(minval, maxval) %>% abs() %>% max()

  c(seq(-abs_maxval, 0, length.out=ceiling(n/2) + 1),
    seq(abs_maxval/n, abs_maxval, length.out=floor(n/2)))
}

CreateForestPlot <- function(x, id_desc_dict = NULL, comparision_labels =
NULL) {
  if(!is.null(id_desc_dict)) {
    x[, "GENESET"] <- id_desc_dict[x[, "GENESET"]]
  }

  fplotdf <- melt(x[, c("GENESET", "NES.x", "NES.y")])
  fplotdf$FDR_raw <- c(x$FDR.x, x$FDR.y)
  fplotdf$FDR <- cut(fplotdf$FDR_raw, breaks = c(0, 0.05, 0.1, 0.2, 0.25),
right = FALSE)
  levels(fplotdf$FDR) <- c("< 0.05", "< 0.1", "< 0.2", "< 0.25"); fplotdf$FDR
%<>% as.character()
  fplotdf$FDR <- factor(fplotdf$FDR, levels = rev(c("< 0.05", "< 0.1", "<
0.2", "< 0.25")), ordered = TRUE)

  fplotdf$GENESET <- factor(fplotdf$GENESET, levels = x[,
"GENESET"][order(rowMeans(x[, c("NES.x", "NES.y")]))])
  if(!is.null(comparision_labels)) {
    levels(fplotdf$variable) <- comparision_labels
  }

  ggplot(fplotdf) +
    geom_point(aes(x = value, y = GENESET, size = FDR)) +
    geom_vline(xintercept = 0) +
    facet_wrap(~variable) +
```

```
    scale_size_discrete(drop = FALSE) +
    xlab("NES") + ylab(NULL)
}
```

Gene set enrichment analysis (Figure 7)

Creating GSEA input files

```
exprdf <-
readxl::read_excel("normalization_geometric_mean_rendezett_oszlopok_2.xlsx",
                             sheet = "normalization_geometric_mean", range =
"A1:X27082", na = "NA")
sampledf <-
readxl::read_excel("normalization_geometric_mean_rendezett_oszlopok_2.xlsx",
                              sheet = "sample description", na = "NA");
sampledf <- sampledf[-1, ]
sampledf$`Sample description (optional)` %<>% gsub("\\+", "", .) %>% gsub("
", "_", .)

colcombs <- list(c(1, 3), c(1, 4), c(5, 7), c(5, 8))
names(colcombs) <- map_chr(colcombs, ~paste(sampledf$`Sample description
(optional)`[.x], collapse = "_vs_"))

dir.create("gsea_inputfiles_220131/")
imap(colcombs, function(.x, .y) {
  tmpdf <- exprdf[, c("gene_symbol", "description", paste0("normalized_S_",
.x))]
  tmpdf[[3]] %<>% as.numeric()
  tmpdf[[4]] %<>% as.numeric()
  colnames(tmpdf) <- c("NAME", "DESCRIPTION", "s1", "s2")
  write.table(tmpdf, file = paste0("gsea_inputfiles_220131/", .y, ".txt"),
quote = FALSE, sep = "\t", row.names = FALSE)
})
```

Running GSEA

Folder *gmxfiles/* contains .gmt files, providing information about gene sets. We downloaded the following files from the official website of GSEA software package (http://www.gsea-msigdb.org/gsea/msigdb/collections.jsp):

- c2.cp.kegg.v7.4.symbols.gmt
- c5.go.bp.v7.4.symbols.gmt
- h.all.v7.4.symbols.gmt

```
gsea_inputs <- list.files("gsea_inputfiles_220131/", full.names = TRUE)
gmx_files <- list.files("gmxfiles/", full.names = TRUE)
gmx_files <- gmx_files[c(4, 8, 12)]

# note: when running GSEA through the PerformGSEA command, you have to
specify the exact location of the files in the directory structure
```

```r
clsfile_path <- "/home/lhgergo/Dokumentumok/LABOR/COPD_GSEA/s1_vs_s2.cls"
resultsdir_path  <-
"/home/lhgergo/Dokumentumok/LABOR/COPD_GSEA/gsea_results_220131/"
input_vars <- expand.grid(gsea_inputs, gmx_files) %>%
set_colnames(c("inputfile", "gmx"))
input_vars$inputfile %<>%
paste0("/home/lhgergo/Dokumentumok/LABOR/COPD_GSEA/", .)
input_vars$gmx %<>% paste0("/home/lhgergo/Dokumentumok/LABOR/COPD_GSEA/", .)
chip_path <-
"/home/lhgergo/Dokumentumok/LABOR/COPD_GSEA/Human_Gene_Symbol_with_Remapping_
MSigDB.v7.4.chip"

pmap(list(input_vars$inputfile, input_vars$gmx),
     ~PerformGSEA(gsea_input = ..1, clsfile = clsfile_path, gmx = ..2,
                  outdir = resultsdir_path, chip = chip_path, gsea_path =
"/home/lhgergo/Programok/GSEA_Linux_4.1.0/gsea-cli.sh"))
```

Creating a catalog of GSEA result directories

```r
resdirs <- list.files("gsea_results_220131/", full.names = TRUE)
paramfiles <- list.files(resdirs, full.names = TRUE) %>%
extract(grepl("\\.rpt", .))

catalog <- sapply(paramfiles, function(crnt_filename) {
  crnt_data <- readLines(crnt_filename) %>% strsplit("\t")
  c(crnt_data[[18]][3] %>% strsplit("\\/") %>% unlist() %>% last() %>%
gsub("\\.txt", "", .),
    crnt_data[[11]][3] %>% strsplit("\\/") %>% unlist() %>% last() %>%
gsub("\\.gmt", "", .))
}) %>% t() %>% as.data.frame() %>% set_colnames(c("comparision",
"geneset_data")) %>% set_rownames(NULL)

catalog$directory <- strsplit(paramfiles, "/") %>% map_chr(~paste0(.x[1:2],
collapse = "/"))
save(catalog, file = "data/results_catalog_220131.RData")
```

Collecting GSEA results

```r
resdirs <- list.files("gsea_results_220131/", full.names = TRUE)

goresults <- lapply(resdirs, function(crnt_resdir) {

  # Loading params
  params <- list.files(crnt_resdir, full.names = TRUE)[grepl("\\.rpt",
list.files(crnt_resdir, full.names = TRUE))] %>%
  readLines() %>% strsplit("\t")
  params <- params[sapply(params, length) == 3] %>% do.call(rbind, .)
  params <- params[, -1]; params <- params[, 2] %>% set_names(params[, 1])

  # Loading XML
  out <- XML::xmlParse(paste0(crnt_resdir, "/edb/results.edb"))
```

```
  rootnode <- xmlRoot(out)

  outdf <- xmlApply(rootnode, xmlAttrs) %>% do.call(rbind.data.frame, .) %>%
    set_colnames(xmlAttrs(rootnode[[1]]) %>% names())
  outdf$RANKED_LIST %<>% gsub("_collapsed_to_symbols.rnk", "", .)
  outdf <- outdf$RANKED_LIST %>% strsplit("_vs_") %>% do.call(rbind, .) %>%
set_colnames(c("sampletype_1", "sampletype_2")) %>% cbind(outdf, .)
  outdf$GENESET %<>%  gsub("gene_sets.gmt#", "", .)
  outdf <- outdf[, c("GENESET", "ES", "NES", "NP", "FDR", "FWER",
"RANK_AT_ES", "RANK_SCORE_AT_ES", "sampletype_1", "sampletype_2")]

  outdf[, c("ES", "NES", "NP", "FDR", "FWER", "RANK_AT_ES",
"RANK_SCORE_AT_ES")] <- outdf[, c("ES", "NES", "NP", "FDR", "FWER",
"RANK_AT_ES", "RANK_SCORE_AT_ES")] %>% lapply(as.numeric)
  outdf$geneset_collection <- params["gmx"] %>% strsplit("\\/") %>%
map_chr(~.x[length(.x)])
  return(outdf)
})

names(goresults) <- map_chr(goresults, ~paste(.x$sampletype_1[1],
.x$sampletype_2[1], .x$geneset_collection[1], sep = "-"))
goresults_signif <- goresults %>% map(~.x[.x$FDR < 0.25, ])
goresults_signif_df <- goresults_signif %>% rbindlist()

save(goresults, goresults_signif, file =
"data/goresults_gsea_standalone_xml_220131.RData")
```

Creating plots

```
load("data/goresults_gsea_standalone_xml_220131.RData")

# creating GSEA comparision dfs ----------
muthits_cm <- rbind(GetMutualHits(goresults_signif$`CD4_CM_T_cell_contol-
CD4_CM_T_cell_adenocarcinoma-h.all.v7.4.symbols.gmt`,
                                  goresults_signif$`CD4_CM_T_cell_contol-
CD4_CM_T_cell_COPD_with_exacerbation-h.all.v7.4.symbols.gmt`),
                    GetMutualHits(goresults_signif$`CD4_CM_T_cell_contol-
CD4_CM_T_cell_adenocarcinoma-c2.cp.kegg.v7.4.symbols.gmt`,
                                  goresults_signif$`CD4_CM_T_cell_contol-
CD4_CM_T_cell_COPD_with_exacerbation-c2.cp.kegg.v7.4.symbols.gmt`))

muthits_em <- rbind(GetMutualHits(goresults_signif$`CD4_EM_T_cell_contol-
CD4_EM_T_cell_adenocarcinoma-h.all.v7.4.symbols.gmt`,
                                  goresults_signif$`CD4_EM_T_cell_contol-
CD4_EM_T_cell_COPD_with_exacerbation-h.all.v7.4.symbols.gmt`),
                    GetMutualHits(goresults_signif$`CD4_EM_T_cell_contol-
CD4_EM_T_cell_adenocarcinoma-c2.cp.kegg.v7.4.symbols.gmt`,
                                  goresults_signif$`CD4_EM_T_cell_contol-
CD4_EM_T_cell_COPD_with_exacerbation-c2.cp.kegg.v7.4.symbols.gmt`))
```

```r
# collecting gene set identifiers ----------
# has to be run once, then you should just load data back from setdf.RData

# genesets_data <- c(readLines("gmxfiles/h.all.v7.4.symbols.gmt"),
#                    readLines("gmxfiles/c2.cp.kegg.v7.4.symbols.gmt"))
#
# setdf <- data.frame(id = genesets_data %>% strsplit("\t") %>%
map_chr(~.x[1]),
#                     url = genesets_data %>% strsplit("\t") %>%
map_chr(~.x[2]))
#
# setdf <- setdf[setdf$id %in% c(muthits_cm$GENESET, muthits_em$GENESET), ]
#
# setdf$description <- pbsapply(setdf$url, function(crnt_url) {
#   crnt_page <- readLines(crnt_url)
#   crnt_page[which(grepl("Brief description", crnt_page)) + 1] %>% trimws()
%>% gsub("</td>", "", .) %>% gsub("<td>", "", .)
# }) %>% set_names(setdf$id)
#
# save(setdf, file = "setdf.RData")

load("setdf.RData")

setdf$description <- paste0(strsplit(setdf$id, "_") %>% map_chr(~.x[1]), ":
", setdf$description)
id_desc_dict <- setdf$description %>% set_names(setdf$id)

# generating background objects for the plots ----------
data_cm <- rbind(GetMutualHits(goresults_signif$`CD4_CM_T_cell_contol-
CD4_CM_T_cell_adenocarcinoma-h.all.v7.4.symbols.gmt`,
                               goresults_signif$`CD4_CM_T_cell_contol-
CD4_CM_T_cell_COPD_with_exacerbation-h.all.v7.4.symbols.gmt`),
                 GetMutualHits(goresults_signif$`CD4_CM_T_cell_contol-
CD4_CM_T_cell_adenocarcinoma-c2.cp.kegg.v7.4.symbols.gmt`,
                               goresults_signif$`CD4_CM_T_cell_contol-
CD4_CM_T_cell_COPD_with_exacerbation-c2.cp.kegg.v7.4.symbols.gmt`))

data_em <- rbind(GetMutualHits(goresults_signif$`CD4_EM_T_cell_contol-
CD4_EM_T_cell_adenocarcinoma-h.all.v7.4.symbols.gmt`,
                               goresults_signif$`CD4_EM_T_cell_contol-
CD4_EM_T_cell_COPD_with_exacerbation-h.all.v7.4.symbols.gmt`),
                 GetMutualHits(goresults_signif$`CD4_EM_T_cell_contol-
CD4_EM_T_cell_adenocarcinoma-c2.cp.kegg.v7.4.symbols.gmt`,
                               goresults_signif$`CD4_EM_T_cell_contol-
CD4_EM_T_cell_COPD_with_exacerbation-c2.cp.kegg.v7.4.symbols.gmt`))

absmax <- c(data_cm$NES.x, data_cm$NES.y, data_em$NES.x, data_em$NES.y) %>%
Rfast::min_max() %>% abs() %>% max()
```

```r
# unifying scales
plt_cm <- CreateForestPlot(data_cm, c("Adenocarcinoma vs. control",
"Exacerbated COPD vs. control"), id_desc_dict = id_desc_dict) +
  scale_x_continuous(limits = c(-absmax, absmax)) +
  scale_y_discrete(labels = function(x) str_wrap(x, width = 40)) +
  ggtitle(label = "") +
  theme_minimal() +
  theme(text = element_text(size = 15),
        panel.spacing.x = unit(3, "lines"),
        panel.background = element_rect(fill = 'white', colour = 'white'),
        plot.background = element_rect(fill = 'white', colour = 'white'),
        axis.text.x = element_text(size = 12, color = "black"),
        axis.text.y = element_text(size = 12, color = "black"))

plt_em <- CreateForestPlot(data_em, c("Adenocarcinoma vs. control",
"Exacerbated COPD vs. control"), id_desc_dict = id_desc_dict) +
  scale_x_continuous(limits = c(-absmax, absmax)) +
  scale_y_discrete(labels = function(x) str_wrap(x, width = 40)) +
  ggtitle(label = "") +
  theme_minimal() +
  theme(text = element_text(size = 15),
        panel.spacing.x = unit(3, "lines"),
        panel.background = element_rect(fill = 'white', colour = 'white'),
        plot.background = element_rect(fill = 'white', colour = 'white'),
        axis.text.x = element_text(size = 12, color = "black"),
        axis.text.y = element_text(size = 12, color = "black"))


plot_grid(plotlist = align_plots(plt_cm, plt_em, align = "v"), ncol = 1,
rel_heights = c(0.3, 0.7),
          labels = c("CD4+ central memory T cells", "CD4+ effector memory T
cells"))
plot_grid(plotlist = align_plots(plt_cm, plt_em, align = "v"), ncol = 1,
rel_heights = c(0.3, 0.7),
          labels = c("CD4+ central memory T cells", "CD4+ effector memory T
cells"))

plot_grid(plotlist = align_plots(plt_cm, plt_em, align = "v"), ncol = 1,
rel_heights = c(0.3, 0.7),
          labels = c("CD4+ central memory T cells", "CD4+ effector memory T
cells")) %>%
  save_plot(filename = "plots/gsea_results_plot.png", plot = ., base_width =
25*1.25, base_height = 17*1.25, units = "cm")
```

Visualizing cytokine levels on a heatmap (Figure 8)

```r
hmdf <- readxl::read_excel("Immuno_onco_merck.xlsx", sheet =
"kiért_goodformat") %>% as.data.frame()
analytes <- hmdf$analyte
```

```r
hmdf$analyte <- NULL
hmdf %<>% as.matrix()
class(hmdf) <- "numeric"
rownames(hmdf) <- analytes

# creating normalized hmdf ----------
hmdf_norm <- apply(hmdf, 1, zscore) %>% t()

# removing Deszk_kontrol2 ----------
hmdf_norm <- hmdf_norm[, !colnames(hmdf_norm) %in% "Deszk_kontrol2"]

# creating annotations ----------
# more detailed annotation of adeno samples
colnames(hmdf_norm)[grepl("_K", colnames(hmdf_norm)) | grepl("_kontrol",
colnames(hmdf_norm))]
catsvctr2 <- c()
catsvctr2[grepl("Koranyi_K", colnames(hmdf_norm))] <- "Healthy control -
smoker"
catsvctr2[grepl("_kontrol", colnames(hmdf_norm))] <- "Healthy control - non-
smoker"
catsvctr2[grepl("_COPD_stabil", colnames(hmdf_norm))] <- "COPD - stable"
catsvctr2[grepl("_COPD_exa", colnames(hmdf_norm))] <- "COPD - exacerbated"
catsvctr2[grepl("_PD", colnames(hmdf_norm))] <- "Adenocarcinoma - PD"
catsvctr2[grepl("_KEM", colnames(hmdf_norm))] <- "Adenocarcinoma -
chemotherapy"
catsvctr2[grepl("_Adeno", colnames(hmdf_norm))] <- "Adenocarcinoma - non-
treated"

annotdf <- data.frame(`Patient category` = catsvctr2) %>%
set_rownames(colnames(hmdf_norm))

# reducing the number of groups to 4 ----------
categories_to_keep <- c("Healthy control - smoker", "COPD - stable", "COPD -
exacerbated", "Adenocarcinoma - non-treated")
annotdf <- annotdf[annotdf$Patient.category %in% categories_to_keep, , drop =
FALSE]

hmdf_norm <- hmdf_norm[, colnames(hmdf_norm) %in% rownames(annotdf)]

# clustering columns ----------
hmdf_cols_dist <- hmdf_norm %>% t() %>% dist(upper = TRUE)
clobj <- hclust(hmdf_cols_dist)
annotdf$Cluster <- clobj %>% cutree(k = 3) %>% extract(rownames(annotdf)) %>%
as.factor()
levels(annotdf$Cluster) <- c(2, 3, 1)
annotdf$Cluster <- paste0("Cluster ", annotdf$Cluster)
colnames(annotdf)[1] <- "Patient category"

annotdf$`Patient category` %<>% factor(levels = categories_to_keep %>% rev())
```

```
##### CREATING PLOTS ###########################################
# preparing colors ----------
n_colors_hmp = 100
colpal_cells <- colorRampPalette(c("#3C5488B2", "white",
"#DC0000B2"))(n_colors_hmp)

full_palette <- pal_npg()(10)
colpal_annots <- list(Cluster = full_palette[1:3] %>%
set_names(paste0("Cluster ", 1:3)),
                      `Patient category` = full_palette[4:7] %>%
set_names(sort(unique(annotdf$`Patient category`))))

# frequency of sample types per clusters ----------
freqsdf <- table(annotdf$`Patient category`, annotdf$Cluster) %>%
as.data.frame() %>%
  set_colnames(c("Patient category", "Cluster", "Freq"))

plt1_horiz <- ggplot(freqsdf, aes(y=Cluster, x=Freq, fill=`Patient
category`)) +
  geom_bar(stat="identity", width=0.6, position = "fill") +
  scale_fill_manual(values = rev(colpal_annots$`Patient category`)) +
  scale_y_discrete(limits = rev(levels(freqsdf$Cluster))) +
  ylab(NULL) + xlab("Relative frequency") +
  theme_minimal() +
  theme(legend.position = "bottom",
        text = element_text(size = 12, color = "black"),
        axis.text.x = element_text(size = 12, color = "black"),
        axis.text.y = element_text(size = 12, color = "black"))

# frequency of sample types per clusters ----------
freqsdf$Cluster_reverse <- factor(freqsdf$Cluster, levels =
rev(levels(freqsdf$Cluster)))
plt2_horiz <- ggplot(freqsdf, aes(y=`Patient category`, x=Freq,
fill=Cluster_reverse)) +
  geom_bar(stat="identity", width=0.6, position = "fill") +
  scale_fill_manual(values = colpal_annots$Cluster) +
  guides(fill = guide_legend(reverse = TRUE)) +
  ylab(NULL) + xlab("Relative frequency") +
  theme_minimal() +
  labs(fill = "Cluster") +
  theme(legend.position = "bottom",
        text = element_text(size = 12, color = "black"),
        axis.text.x = element_text(size = 12, color = "black"),
        axis.text.y = element_text(size = 12, color = "black"))

# creating the heatmap
hmpobj <- pheatmap::pheatmap(hmdf_norm, annotation_col = annotdf,
                             color = colpal_cells,
```

```
                                   cutree_cols = 3,
                                   annotation_colors = colpal_annots,
                                   breaks = CreateBreaks2(minval = min(hmdf_norm),
maxval = 4, n = n_colors_hmp),
                                   treeheight_row = 0, show_colnames = FALSE,
legend = TRUE,
                                   annotation_legend = FALSE, cluster_cols = clobj,
fontsize = 12)

###### CREATING THE COMPOSITE PLOT ----------
library(gridExtra)
plot_grid(hmpobj$gtable,
          plot_grid(plt1_horiz + guides(fill = guide_legend(nrow = 4, byrow =
TRUE)),
                    plt2_horiz, nrow = 1, labels = c("B", "C")), nrow = 2,
labels = "A") %>%
  save_plot(filename = "plots/hmplot_4grps.png", plot = ., base_width =
25*1.25, base_height = 17*1.25, units = "cm")
```
