# Supplementary material for: Single-cell immunophenotyping revealed the association of CD4+ central and CD4+ effector memory T cells linking exacerbating chronic obstructive pulmonary disease and NSCLC
Source: Front Immunol. 2023 Dec 20;14:1297577. doi: 10.3389/fimmu.2023.1297577 (PMC10770259; doi:10.3389/fimmu.2023.1297577)
Supplement: Supplementary file 5 [file Table_4.pdf]

Short name of the protein	Full name of the protein	Alternative names of the protein	Gene name	UniProt ID	Detection range pg/ml
BTLA	B- and T-lymphocyte attenuator	B- and T-lymphocyte-associated protein, CD272	BTLA	Q7Z6A9	16.63-198,560
CD27	CD27 antigen	Tumor necrosis factor receptor superfamily member 7	CD27	P26842	10.04-172,511
CD28	T-cell-specific surface glycoprotein CD28	TP44	CD28	P10747	1121.23-199,220
CD40	Tumor necrosis factor receptor superfamily member 5	CD40L receptor	CD40	P25942	2.37-23,003
CD80/B7-1	T-lymphocyte activation antigen CD80	Activation B7-1 antigen	CD80	P33681	7.59-71,188
CD86/B7-2	T-lymphocyte activation antigen CD86	Activation B7-2 antigen	CD86	P42081	32.56-186,951
CTLA-4	Cytotoxic T-lymphocyte protein 4	Cytotoxic T-lymphocyte-associated antigen 4, CD152	CTLA4	P16410	5.04-74,684
GITR	Tumor necrosis factor receptor superfamily member 18	Glucocorticoid-induced TNFR-related protein, CD357	TNFRSF18	Q9Y5U5	3.07-79,814
GITRL	Tumor necrosis factor ligand superfamily member 18	Glucocorticoid-induced TNF-related ligand	TNFSF18	Q9UNG2	13.57-29,449
HVEM	Tumor necrosis factor receptor superfamily member 14	Herpes virus entry mediator A, CD270	TNFRSF14	Q92956	1.22-14,501
ICOS	Inducible T-cell costimulator	Activation-inducible lymphocyte immunomediatory molecule, CD278	ICOS	Q9Y6W8	23.79-198,972
LAG-3	Lymphocyte activation gene 3 protein	CD223	LAG3	P18627	57.66-198,219
PD-1	Programmed cell death protein 1	CD279	PDCD1	Q15116	14.51-149,468
PD-L1	Programmed cell death 1 ligand 1	CD274	CD274	Q9NZQ7	2.37-36,458
PD-L2	Programmed cell death 1 ligand 2	CD273	PDCD1LG2	Q9BQ51	15.85-189,115
TIM-3	Hepatitis A virus cellular receptor 2	T-cell immunoglobulin mucin receptor 3, CD366	HAVCR2	Q8TDQ0	2.33-36,013
TLR-2	Toll-like receptor 2	Toll/interleukin-1 receptor-like protein 4, CD282	TLR2	O60603	33.88-198,927

Supplementary Table 4. The list of the proteins measured in the plasma of the human subjects enrolled in the study.
